# Does the endometrial cavity have a molecular microbial signature?

**DOI:** 10.1038/s41598-019-46173-0

**Published:** 2019-07-09

**Authors:** Andrew D. Winters, Roberto Romero, Maria Teresa Gervasi, Nardhy Gomez-Lopez, Maria Rosa Tran, Valeria Garcia-Flores, Percy Pacora, Eunjung Jung, Sonia S. Hassan, Chaur-Dong Hsu, Kevin R. Theis

**Affiliations:** 1 Department of Biochemistry, Microbiology, and Immunology, Wayne State University School of Medicine, Detroit, Michigan USA; 2 Perinatal Research Initiative in Maternal, Perinatal and Child Health, Wayne State University School of Medicine, Detroit, Michigan USA; 3 Perinatology Research Branch, Division of Obstetrics and Maternal-Fetal Medicine, Division of Intramural Research, Eunice Kennedy Shriver National Institute of Child Health and Human Development, National Institutes of Health, U.S. Department of Health and Human Services, Bethesda, MD, and Detroit, MI, Detroit, Michigan USA; 4 Department of Obstetrics and Gynecology, University of Michigan, Ann Arbor, Michigan USA; 5 Department of Epidemiology and Biostatistics, Michigan State University, East Lansing, Michigan USA; 6 Center for Molecular Medicine and Genetics, Wayne State University, Detroit, Michigan USA; 7 Detroit Medical Center, Detroit, Michigan USA; 8 Department of Women’s and Children’s Health, University Hospital of Padua, Padua, Italy; 9 Department of Obstetrics and Gynecology, Wayne State University School of Medicine, Detroit, Michigan USA; 10 Department of Physiology, Wayne State University School of Medicine, Detroit, Michigan USA

**Keywords:** Applied microbiology, Clinical microbiology, Microbiome, Microbiology techniques, High-throughput screening

## Abstract

Recent molecular studies concluded that the endometrium has a resident microbiota dominated by *Lactobacillus* spp. and is therefore similar to that of the vagina. These findings were largely derived from endometrial samples obtained through a transcervical catheter and thus prone to contamination. Herein, we investigated the molecular microbial profiles of mid-endometrial samples obtained through hysterectomy and compared them with those of the cervix, vagina, rectum, oral cavity, and controls for background DNA contamination. Microbial profiles were examined through 16S rRNA gene qPCR and sequencing. Universal bacterial qPCR of total 16S rDNA revealed a bacterial load exceeding that of background DNA controls in the endometrium of 60% (15/25) of the study subjects. Bacterial profiles of the endometrium differed from those of the oral cavity, rectum, vagina, and background DNA controls, but not of the cervix. The bacterial profiles of the endometrium and cervix were dominated by *Acinetobacter*, *Pseudomonas*, *Cloacibacterium*, and Comamonadaceae. Both 16S rRNA gene sequencing and *Lactobacillus* species-specific (*L*. *iners* & *L crispatus*) qPCR showed that *Lactobacillus* was rare in the endometrium. In conclusion, if there is a microbiota in the middle endometrium, it is not dominated by *Lactobacillus* as was previously concluded, yet further investigation using culture and microscopy is necessary.

## Introduction

The endometrium has traditionally been considered sterile in non-pregnant women, in pregnant women, and in the puerperium based upon cultivation studies^1–16^. Indeed, infection of the endometrium or the presence of high concentrations of microbial products (e.g. endotoxin) has been associated with implantation failure^17,18^, spontaneous abortion^19^, recurrent miscarriage^20^, and spontaneous preterm birth^21,22^.

It is difficult to envision that the mucosa of the endometrial cavity could be continuously exposed to microorganisms present in the lower genital tract, as well as to sperm that can potentially carry microorganisms into the uterus, and yet remain free of bacterial colonization^22^. Recent studies using molecular techniques suggest that there may be a resident microbiota (i.e., the assemblage of microorganisms present in a defined environment^23^) in the human endometrium^24–37^, and the potential relevance of these microbes to women’s reproductive health, especially fertilization and normal pregnancy outcomes, is being considered^26,28,38^. For instance, *Lactobacillus*-dominance (>90% relative abundance) of the endometrial microbiota has been associated with implantation success^28,34^ and live birth rates in women undergoing *in vitro* fertilization (IVF)^28^. It has therefore been suggested that an increase in relative abundance of *Lactobacillus* species to >90% in women with a non-*Lactobacillus*-dominated endometrial microbiota might promote implantation success among infertile patients^34^.

Nevertheless, a fundamental question remains as to whether an endometrial microbiota truly exists^39^. The potential limitations of previous reports are two-fold. First, most of the previous molecular surveys investigating an endometrial microbiota analyzed samples that had been collected transcervically (11/15 studies in Supplementary Table 1); this sampling approach is prone to contamination of the endometrium with microbes and/or microbial molecular signals from the vagina and cervix. Indeed, a culture-based investigation of endometrial bacteria demonstrated an increased rate of bacterial isolation from samples obtained transcervically than transabdominally^10^. As a result, the widely reported *Lactobacillus*-dominance of the endometrial microbiota may be due to the influence of contamination with vaginal *Lactobacillus* species during sampling. A second reason further investigation is necessary is that, if an endometrial microbiota truly exists, then it is present at very low biomass and thus its molecular characterization is susceptible to influences of background DNA contamination from extraction kits and PCR and sequencing reagents (collectively referred to as the “kitome”)^40–44^. As a result, contaminating DNA may constitute a considerable portion, if not all, of the observed molecular microbial signatures within the endometrium. It is therefore necessary that molecular investigations of an endometrial microbiota incorporate technical controls for potential sources of background DNA contamination and provide detailed descriptions of the microbial profiles of these controls when characterizing the endometrial microbiota. To date, the majority of sequence-based molecular surveys investigating an endometrial microbiota either have not incorporated technical controls or have not provided detailed descriptions of the microbial profiles of these controls (10/14 sequence-based studies in Supplementary Table 1).

Given the potential of both the sample collection method and background DNA contamination to shape characterizations of endometrial microbiota profiles, the existence of a resident endometrial microbiota, and its structure if indeed present, remains unknown^39^. The objective of this study was to use 16S rRNA gene sequencing, universal bacterial 16S rDNA qPCR, and *Lactobacillus*-targeted qPCR to characterize the bacterial profiles of endometrial samples from non-pregnant women who underwent transabdominal hysterectomy. The study further determined if the endometrial microbiota is distinct from that of other body sites by comparing the bacterial profiles of the endometrium to those of the cervix, vagina, rectum, and oral cavity of the women, as well as to technical controls for potential background DNA contamination.

## Materials and Methods

### Study population

This was a cross-sectional study of 25 women with a median age of 45 years (IQR: 41–49.5) who underwent a hysterectomy, primarily for fibroids (23/25), at the Azienda Ospedaliera di Padova, Italy. The two women without fibroids underwent hysterectomy for endometrial hyperplasia. Exclusion criteria included antibiotic administration within the last 10 days, vaginal bleeding, vaginal douching, the use of intrauterine contraceptive devices, and digital examinations with antimicrobial agents. Additionally, women undergoing laparoscopic hysterectomy were not eligible to participate. Protocols were approved by the University of Padua. Methods were carried out in accordance with the relevant guidelines and regulations. Written informed consent was obtained from all participating women on or before admission.

### Sample collection

Cervical, vaginal, rectal, and oral Dacron swabs were obtained from each woman within 24 hours of the hysterectomy procedure. During a speculum examination, a swab was gently rotated around the external cervical os (i.e., the opening of the cervical canal into the vagina) for approximately 30 seconds and then removed while avoiding contact with vaginal structures. A second swab was then used to collect vaginal fluid from the posterior fornix, again allowing 30 seconds for saturation of the swab. Transabdominal hysterectomy was performed, with intraoperative antibiotic prophylaxis not being administered until the uterus had been removed. Upon removal of the uterus, it was handled by a research nurse under sterile conditions in the operating room. A sterile knife was used to separate the cervix from the uterine corpus. The uterus was opened with sterile scissors at 3 and 9 o’clock, taking precautions to avoid contamination of the endometrium. Once the uterus was opened, a Dacron swab was collected from the middle portion of the endometrium, an area unlikely to be contaminated during the procedure of opening the uterus, by gently rotating and allowing for saturation of the swab. An analysis was performed on a subset of subjects for whom swabs of both the mid-endometrium and the whole-length endometrium were collected (N = 9). There were no differences in 16S rRNA gene abundance, alpha diversity, or beta diversity between these samples (Supplementary Methods). Therefore, the data presented in this study are from mid-endometrium samples. All swabs were placed in cryovials and frozen at −80 °C until analysis.

### Extraction of DNA from samples

Genomic DNA was extracted from swab samples using a QIAGEN DNeasy PowerLyzer PowerSoil Kit with minor modifications to the manufacturer’s protocol. Within the supplied bead tube, swabs were immersed in 500 μl of bead solution and 200 μl of phenol:chloroform:isoamyl alcohol (pH 7–8) solution for 10 minutes. Sixty μl of Solution C1 were added, and microbial cells were lysed by mechanical disruption using a bead beater (twice at 30 seconds each). The bead tubes were centrifuged, and the supernatants were transferred to new tubes. Next, 100 μl of solution C2, 100 μl of solution C3, and one μl of RNase A enzyme were added and incubated at 4 °C for five minutes. Steps involving solutions C2 and C3 were combined to maximize DNA yield. Tubes were centrifuged, and supernatants were transferred to new tubes that contained 650 μl of solution C4 and 650 μl of 100% ethanol. The lysates were loaded onto filter columns, centrifuged for one minute, and the flow-through was discarded. This step was repeated until all sample lysates were spun through the filter columns. Five hundred μl of solution C5 were added to the filter columns, centrifuged for one minute, the flow-through was discarded, and the tube was centrifuged for an additional two minutes as a dry-spin. Finally, 60 μl of solution C6 were placed on the filter column and incubated for five minutes before centrifuging for 30 seconds to elute the extracted DNA. For each set of extractions, one blank DNA extraction kit was processed as a background negative control. Purified DNA was stored at −20 °C.

### Quantitative real-time PCR (qPCR) of the 16S rRNA gene in samples

Three qPCR assays targeting the 16S rRNA genes in samples were performed. Total bacterial DNA abundance within samples was measured via amplification of the V1 - V2 region of the 16S rRNA gene according to the protocol of Dickson *et al*.^45^ with minor modifications. These modifications included the use of a degenerative forward primer (27f-CM: 5′-AGA GTT TGA TCM TGG CTC AG-3′)^46^ and a degenerate probe containing locked nucleic acids (+) (BSR65/17: 5′-56FAM-TAA +YA+C ATG +CA+A GT+C GA-BHQ1-3′). Each 20 μl reaction contained 0.6 μM of 27f-CM primer, 0.6 μM of 357 R primer (5′-CTG CTG CCT YCC GTA G-3′), 0.25 μM of BSR65/17 probe, 10.0 μl of 2X TaqMan Environmental Master Mix 2.0 (Life Technologies, Carlsbad, CA), and 4.0 μl purified DNA. The total bacterial DNA qPCR was performed under the following conditions: 95 °C for 10 min, followed by 40 cycles of 94 °C for 30 sec, 57 °C for 30 sec, and 72 °C for 30 sec.

Absolute abundances of *Lactobacillus iners* and *L*. *crispatus* were measured within a subset of vaginal, cervical, and endometrial samples from 10 and 8 women, respectively. Samples from these women were selected because the relative abundance of *L*. *iners* or *L*. *crispatus* in the bacterial profile of their vaginal sample was representative of the spectrum of relative abundances (~ 0–100%) of these bacteria observed among vaginal samples overall. Absolute abundances were measured via qPCR assays using taxon-specific primers described by Fredricks *et al*.^47^ (*L*. *crispatus*: 5′-TCT TGA CAT CTA GTG CCA TTT GT-3′ and 5′-TGC ACC ACC TGT CTT AGC-3′) and Srinivasan *et al*.^48^ (*L*. *iners*: 5′-GAT GCT AAT ACC GGA TAA YAA CAG AT-3′ and 5′-CAC CGC AGG TCC ATC CAA GA-3′). For both assays, each 20 μl reaction contained 1.0 μM of each primer, 10.0 μl of 2X Powerup SYBR Green Master Mix (Thermo Fisher Scientific, Carlsbad, CA), and 2.0 μl purified DNA. Cycling conditions for the *L*. *iners* assay were: 95 °C for 10 min, and 45 cycles of 94 °C for 30 sec, 57 °C for 30 sec, and 72 °C for 30 sec. Cycling conditions for the *L*. *crispatus* assay were: 95 °C for 2 min, and 45 cycles of 95 °C for 15 sec and 60 °C for 1 min. For the taxon-specific qPCR assays, all samples were run in duplicate in a single run (total of two runs).

**Figure 1:**
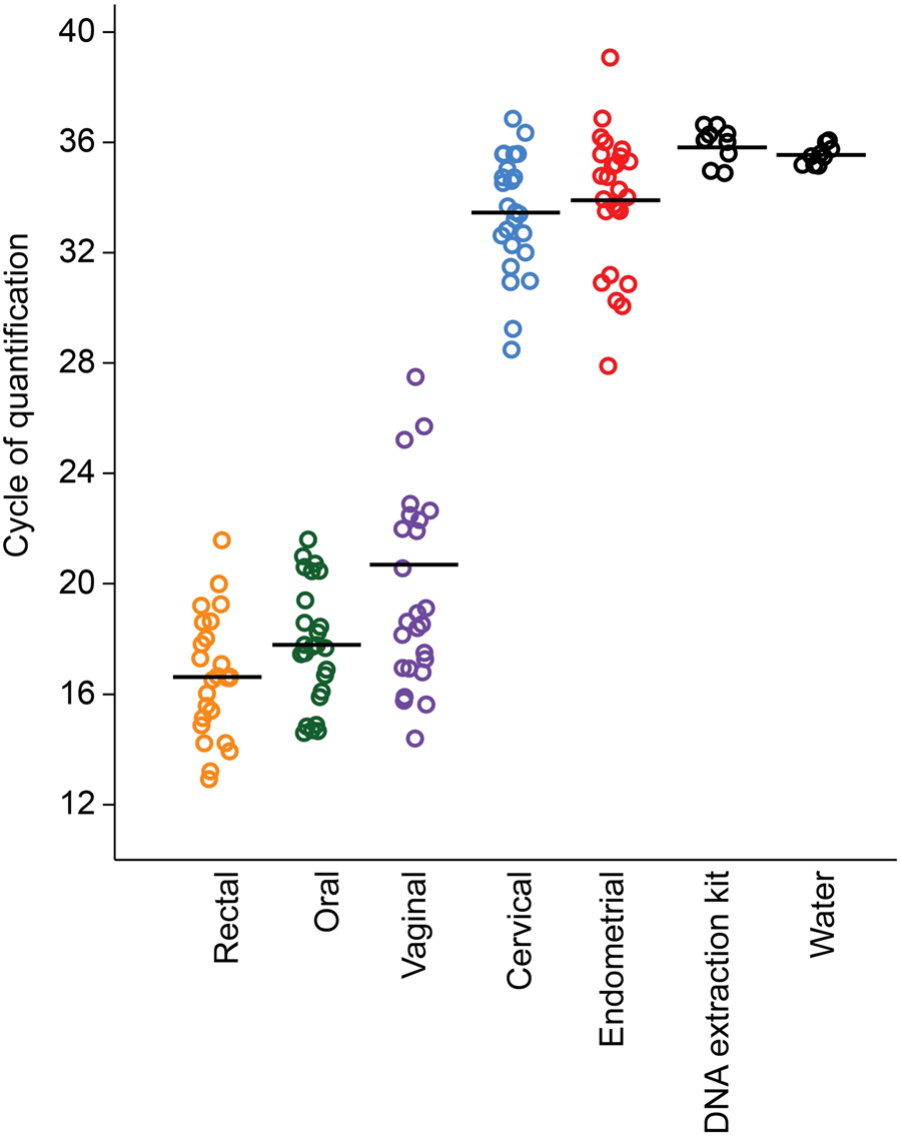
Quantitative real-time PCR (qPCR) analysis illustrating differences in 16S rRNA gene abundance based on cycle of quantification (Cq) values among oral, rectal, vaginal, cervical, endometrial, and technical control samples, including nuclease-free water. Bars indicate mean values.

For all qPCR assays, raw amplification data were normalized to the ROX passive reference dye and analyzed with Standard Curve 3.3.0-SR2-build15 (Thermo Fisher Cloud), using automatic threshold and baseline settings. Cycle of quantification (Cq) values were calculated for samples based on the mean number of cycles required for normalized fluorescence to exponentially increase. In an effort to limit analyses to samples that produced bacterial signals beyond those evident in background technical controls, only samples of body sites that produced Cq values less than 35 for the total 16S rRNA gene qPCR (V1-V2) analysis were included in downstream 16S rRNA gene sequence-based analyses **(**Fig. 1**)**.

To assess differences in 16S rDNA abundance between endometrial, cervical, vaginal, rectal, and oral samples among the 25 subjects, differences in qPCR Cq values were evaluated via repeated measures ANOVA followed by Tukey’s pairwise comparisons^49,50^. *F*-tests were used to evaluate unequal variances in Cq values between body sites and background technical control samples. To assess differences in 16S rDNA abundance between individual body sites and background technical control samples, *t*-tests or Mann-Whitney *U*-tests were used. Statistical analyses were performed using PAST software (v2.17c)^51^.

### Sequencing of the 16S rRNA gene in samples

The V4 region of the 16S rRNA gene was selected for analysis because it is an established marker for broad-level phylogenetic analysis of bacterial taxa and it is the region of the 16S rRNA gene most representative of the nearly full-length gene^52^. Sequencing of the V4 region has been previously used to profile the bacterial communities of multiple human body sites, including the skin^53^, mouth^54^, lung^55^, gut^56^, vagina^57^, bladder^58^, placenta^59^, and endometrium^25,33^. Amplification and sequencing of the V4 region of the 16S rRNA gene was performed at the University of Michigan’s Center for Microbial Systems (Ann Arbor, MI) using the dual indexing sequencing strategy developed by Kozich *et al*.^60^. Sequencing was conducted using the Illumina MiSeq platform (V2 500 cycles, Illumina MS102-2003), according to the manufacturer’s instructions with modifications found in Kozich *et al*.^60^. Each PCR reaction contained 1.0 µM of each primer, 5.0 µl template DNA, 0.15 µl AccuPrime HiFi Polymerase, and DNase-free water to produce a final volume of 20 µl. Standard PCR was performed using the following conditions: 95 °C for 2 minutes, followed by 30 cycles of 95 °C for 20 seconds, 55 °C for 30 seconds, and 72 °C for 5 minutes, with an additional elongation at 72 °C for 10 minutes.

The resulting fragments were visualized in 3% agarose gel. Several cervical, endometrial, and blank DNA extractions kit samples produced no or weak bands, indicating that sequencing of these samples was unlikely to yield sufficient sequence data for downstream analysis. Therefore, DNA amplification was additionally performed on cervical, endometrial, and technical control samples using touchdown PCR. This method reduces the initial amplification of nonspecific host DNA sequences during early steps of amplification by using a relatively high primer annealing temperature in relation to the melting point of the primers, and incrementally decreasing the annealing temperature as cycling proceeds^61^. This method has been recently used to investigate the microbiota of several body sites with low microbial biomass, including the lung^62,63^ and brain^64^.

Touchdown PCR was performed using the following conditions: 2 min at 95 °C, followed by 20 cycles of 95 °C for 20 s, 60 °C for 15 s, and 72 °C for 5 min (with a 0.3 °C decrease of the 60 °C annealing temperature each cycle), followed by 20 cycles of 95 °C for 20 s, 55 °C for 15 s, and 72 °C for 5 min, followed by 72 °C for 10 min. Three µl of template DNA were included in each touchdown PCR reaction. Sequencing libraries were prepared according to Illumina’s protocol for Preparing Libraries for Sequencing on the MiSeq (15039740 Rev. D) for 2 nM or 4 nM libraries. FASTQ files were generated for paired-end reads. Sample-specific MiSeq run files have been deposited on the NCBI Sequence Read Archive (BioProject ID PRJNA543861).

### 16S rRNA gene sequence processing and data analysis

Prior to analyzing bacterial profile alpha and beta diversity, raw sequenced reads were processed using Mothur software (v1.39.5)^65^, following the Standard Operating Procedure provided by Schloss *et al*. (www.mothur.org/wiki/MiSeq_SOP). Paired-end reads were assembled into contiguous sequences, quality checked (maximum length = 300, maximum ambiguous base pairs = 0, and maximum number of homopolymers = 8), and aligned against the SILVA 16S rDNA reference database (release 102); sequences falling outside the target alignment space were removed^66,67^. Quality sequences were pre-clustered (diffs = 2) and chimeric sequences were identified with VSEARCH and removed^68^. The remaining sequences were taxonomically classified using the SILVA reference database^67^ with a k-nearest neighbor approach and a confidence threshold of 80%. Sequences derived from an unknown domain, Eukaryota, Archaea, chloroplasts, or mitochondria were removed. Operational taxonomic units (OTUs) were defined by clustering sequences at a 97% sequence similarity cutoff using the average neighbor method. Sequencing of mid-endometrial, whole-length endometrial, cervical, vaginal, rectal, oral, and background technical control samples yielded 5,715,294 sequences. They clustered into 3,338 OTUs (1,019 singletons, and 517 doubletons). The median number of sequences per sample was 16,676 (13,026–20,906 95% CI). The raw OTU data from this study are provided as Supplementary Data.

Heatmaps were generated for prominent OTUs (i.e., average relative abundance ≥1%) using the open-source software program Morpheus (https://software.broadinstitute.org/morpheus). For determining the taxonomic identities of select OTUs beyond the genus level, resultant consensus sequences were submitted for Basic Local Alignment Search Tool (BLAST)^69^ analysis. To investigate linear relationships between absolute 16S rDNA abundances and the relative abundances of individual bacterial taxa in sequencing surveys, Spearman’s correlation tests were used.

Alpha diversity analyses were based on three metrics: Chao 1 richness estimator, Shannon diversity index, and the inverse Simpson index, each calculated using Mothur software (v1.39.5)^65^. Differences in alpha diversity values between body sites and background technical controls samples were evaluated using Mann-Whitney *U*-tests in R (v3.5.1)^70^. Differences in alpha diversity values among paired body site samples were evaluated using linear mixed-effect models and ANOVA tests, controlling for subject (i.e. patient) identity as a random effect using the R-package lme4^71^, followed by Tukey’s pairwise comparisons with Bonferroni adjustments using the R-package multcomp^72^.

Beta diversity was assessed using Jaccard and Bray-Curtis similarity indices to reflect bacterial profile composition and structure, respectively. Similarity values were calculated using percent relative abundance data for OTUs within samples. Beta diversity was visualized through Principal Coordinates Analyses (PCoA), and statistically evaluated using PERMANOVA with 9,999 permutations^73^. The influences of subject identity and body site in structuring the microbiota were concurrently investigated using the adonis function in the R-package vegan (v2.4-6)^74^. Linear discriminant analysis effect size, or LEfSe^75^, was used with default parameters (α = 0.05 and LDA score 2.0) to identify any OTUs that differed in relative abundance between body sites and/or background technical controls. Prior to LEfSe analysis, singleton and doubleton OTUs were removed from the dataset.

Analysis of the bacterial profiles of body site samples was performed only on samples with a qPCR Cq value less than 35 (all blank DNA extraction kit controls had a Cq value ≥ 34.9). Additionally, analyses were limited to body site samples that had Good’s coverage values greater than 98% and contained at least 500 (standard PCR dataset) or 1,000 (touchdown PCR dataset) sequences. For analyses of alpha diversity, sequence libraries were subsampled to a depth of either 500 or 1,000 sequences, for the standard PCR and touchdown PCR data sets, respectively. For beta diversity analyses, no subsampling was performed. Background technical controls were included in beta diversity analyses only if they had Good’s coverage values greater than 90% and yielded at least 100 quality sequences.

A *post*-*hoc* analysis was conducted on the 16S rRNA gene sequences from cervical, endometrial, and technical control samples using the Dada2 (version 1.8) package^76^ in R (v3.5.1)^70^, and the online MiSeq protocol (https://benjjneb.github.io/dada2/tutorial.html). This secondary analysis of amplicon sequence variants (ASVs), defined by 100% sequence similarity, may afford higher resolution of prominent sequence variants among body site and technical control samples. Amplicon sequence variant analysis was only performed on cervical and endometrial samples with a qPCR Cq value less than 35, and which had at least 200 sequences with a Good’s coverage value > 90% for the standard PCR dataset, or at least 420 sequences and a Good’s coverage value > 97% for the touchdown PCR dataset. Amplicon sequence variant analysis was performed on all background DNA control samples.

## Results

### Absolute 16S rDNA abundance (i.e. bacterial load) among body sites

Quantitative real-time PCR revealed variation in 16S rDNA abundance across body sites **(**Fig. 1**)**. Oral, rectal, and vaginal samples had lower cycle of quantification (Cq) values (i.e. higher bacterial loads) than cervical and endometrial samples (repeated measures ANOVA: df = 124, *F* = 313.7, *p* < 0.0001; Tukey’s pairwise comparisons: *p* = 0.0001). The median fold change in Cq value in vaginal samples compared to cervical and endometrial samples was 1.75 (1.60–1.89 95% CI) and 1.77 (1.62–1.90 95% CI), respectively. Cervical and endometrial samples did not differ in Cq value (*t* = 0.69; *p* = 0.49), but each had a lower Cq value than background technical controls (*p* ≤ 0.02) **(**Fig. 1**)**. Nevertheless, the Cq values of cervical and endometrial samples were variable, with only some (33/50) of these samples exhibiting bacterial molecular signals exceeding those of technical controls **(**Fig. 1**)**. Specifically, the range of Cq values for blank DNA extraction kit controls was 34.9–36.6 cycles; 72% (18/25) of cervical and 60% (15/25) of endometrial samples had Cq values lower than those of extraction controls. Bacterial load in the vagina was not correlated with bacterial load in either the cervix (R^2^ = 0.02, *p* = 0.46) or the endometrium (R^2^ < 0.0001, *p* = 0.99). However, bacterial load in the cervix was positively correlated with that in the endometrium (R^2^ = 0.32, *p* = 0.003).

**Figure 2:**
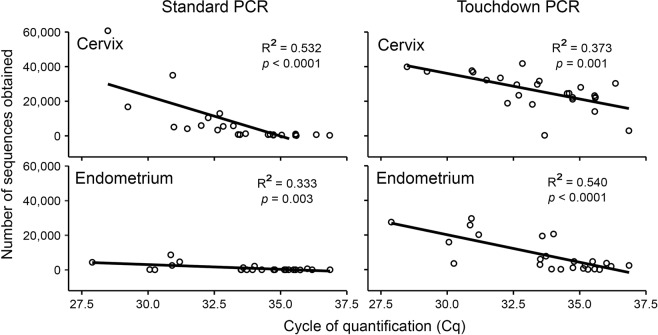
Regressions of the number of quality 16S rRNA gene bacterial sequences obtained from the cervix and endometrium using standard PCR and touchdown PCR approaches against the absolute abundance of 16S rDNA in samples based on cycle of quantification (Cq) values from universal bacterial quantitative real-time PCR.

For the cervix and endometrium, significant correlations were observed between 16S rDNA abundances in the samples, as determined by qPCR, and in the number of quality trimmed bacterial sequences obtained from MiSeq sequencing using both standard PCR and touchdown PCR approaches for generating sequence libraries **(**Fig. 2**)**. These results demonstrate that the number of quality sequences obtained was directly related to the absolute abundances of 16S rDNA in samples, providing confidence that the sequence data from cervical and endometrial samples were not simply due to background DNA contamination (i.e. the “kitome”).

### Bacterial profiles among body sites (standard PCR dataset)

In general, rectal and oral samples had higher levels of taxonomic richness (Chao1 estimator) and heterogeneity (Shannon and Inverse Simpson indices) than vaginal, cervical, and endometrial samples (Supplementary Fig. S1, Table S2). Specifically, the richness and heterogeneity of rectal samples exceeded those of all other sample types. Additionally, the heterogeneity of oral samples was greater than that of vaginal, cervical, and endometrial samples, and the richness of oral samples was greater than that of vaginal and cervical samples, but not that of endometrial samples. Lastly, the richness and heterogeneity of vaginal, cervical, and endometrial samples did not vary, with the exception of cervical samples having higher Shannon diversity indices than vaginal samples (Supplementary Fig. S1, Table S2).

**Figure 3:**
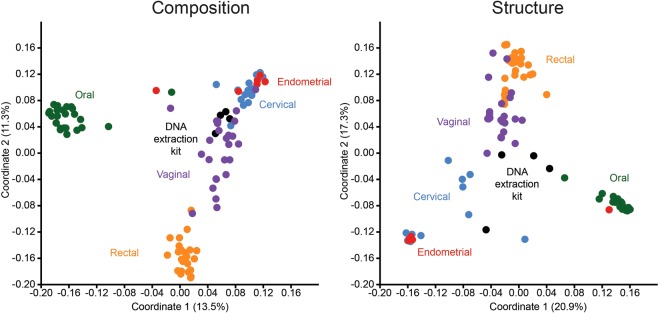
Principal Coordinates Analyses (PCoA) illustrating differences in 16S rRNA gene profiles among oral, rectal, vaginal, cervical, endometrial, and DNA extraction kit samples using a standard PCR approach. Profiles were generated for 16S rRNA gene community composition and structure using the Jaccard and Bray-Curtis indices, respectively.

With respect to beta diversity, there was a high degree of separation among the bacterial profiles of body sites based on community composition (Jaccard Similarity Index; *F* = 9.49, *p* = 0.0001) and structure (Bray-Curtis Similarity Index; *F* = 19.20, *p* = 0.0001) (Fig. 3). Pairwise analyses with sequential Bonferroni corrections showed that, with the exception of the cervix and endometrium (whose profiles did not consistently differ), the bacterial profiles of the body sites differed in both composition and structure (*p* = 0.0001). Although the bacterial profiles of the cervix and endometrium did not differ from one another, the profiles of each differed from those of background technical controls in both composition (*p* ≤ 0.005) and structure (*p* ≤ 0.02) (Fig. 3). When only subjects with paired vaginal, cervical, and endometrial samples that met the cutoff criteria (500 sequences and Good’s coverage ≥98%) were considered (N = 6), and after controlling for subject (i.e. patient) identity, the effect of body site remained for community composition (Jaccard: subject, R^2^ = 0.301, *p* = 0.017; sample type, R^2^ = 0.127, *p* = 0.005), and for community structure (Bray-Curtis: subject, R^2^ = 0.293, *p* = 0.085; sample type, R^2^ = 0.275, *p* = 0.001).

**Figure 4:**
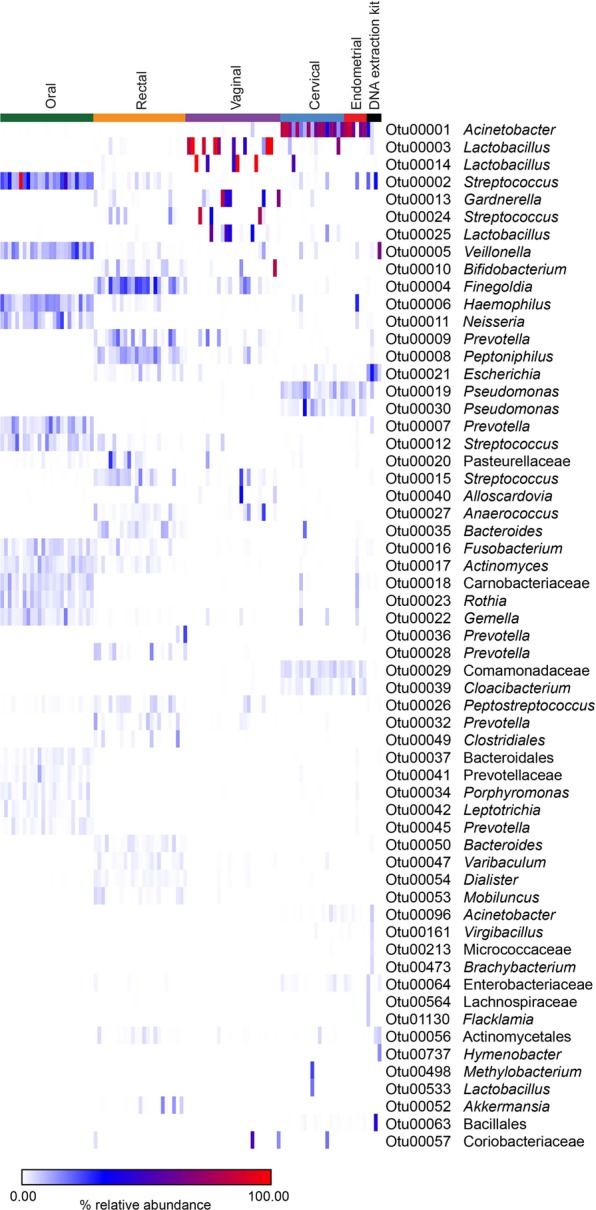
Heat map illustrating percent relative abundances of prominent operational taxonomic units (≥1% average relative abundance) among oral, rectal, vaginal, cervical, endometrial, and DNA extraction kit samples. Amplification of 16S rRNA genes was performed using a standard PCR approach.

Investigation of the taxonomic identities of prominent OTUs (defined as OTUs with ≥ 1% average relative abundance) by sample type revealed overlap in the profiles of cervical and endometrial samples compared with those of vaginal, rectal, and oral samples (Fig. 4). The profiles of vaginal samples were dominated by *Lactobacillus* and *Gardnerella*. The most prominent taxa in rectal samples were *Finegoldia*, *Prevotella*, *Peptoniphilus*, *Streptococcus*, and *Bacteroides*. In oral samples, the most prominent taxa were *Streptococcus*, *Veillonella*, *Haemophilus*, *Neisseria*, *Prevotella*, *Fusobacterium*, *Actinomyces*, *Rothia*, and *Gemella*. In contrast, the bacterial profiles of both cervical and endometrial samples were dominated by *Acinetobacter*, with a single prominent OTU (OTU 1) having average relative abundances of 53.3% in the cervix and 60.6% in the endometrium. Core OTUs (defined as OTUs present in ≥50% of samples, and with a mean relative abundance ≥1%) in both the cervix and endometrium included *Acinetobacter* (OTU 1), *Pseudomonas* (OTUs 19 and 30), Comamonadaceae (OTU 29), and *Cloacibacterium* (OTU 39). Notably, *Lactobacillus* (OTU 3) was a core OTU in the cervix but not in the endometrium. The two most abundant *Lactobacillus* OTUs in the dataset (OTUs 3 and 14) accounted for 26,462/137,551 (19.24%) sequences among cervical samples, and yet only 4/23,754 (0.017%) sequences among endometrial samples.

A BLAST analysis (i.e. comparison of a 16S rRNA gene sequence to those in the BLAST taxonomy database) revealed that the consensus sequence for OTU 1 was identical to that of eight *Acinetobacter* type strains within seven species that have been isolated from clinical samples: *A*. *bereziniae*, *A*. *colistiniresistens*, *A*. *gyllenbergii*, *A*. *junii*, *A*. *modestus*, *A*. *proteolyticus*, *and A*. *vivianii*. A BLAST analysis of OTU 30 showed identical matches for multiple strains of *Pseudomonas aeruginosa*, and an analysis of OTU 19 revealed identical matches for multiple *Pseudomonas* species within the *Pseudomonas putida* group (*P*. *monteilii*, *P*. *plecoglossicida*, *P*. *putida*, *and P*. *taiwanensis*)^77^. A BLAST analysis of OTU 39 showed identical matches for six type strains within the species *Cloacibacterium normanense*, *C*. *caeni*, and *C*. *rupense*. Lastly, a BLAST analysis of OTU 29 (a member of the family Comamonadaceae) revealed identical matches for *Comamonas jiangduensis* and *C*. *kerstersii*.

The most prominent taxa in background technical controls were *Veillonella* (OTU 5), *Escherichia* (OTU 21), *Streptococcus* (OTU 2), and *Acinetobacter* (OTU 1) (Fig. 4). However, *Escherichia* (OTU 21) was the only genus consistently detected in high relative abundances across the technical control samples (12.4% to 31.3%, in three of four controls).

### Bacterial profiles of the cervix and endometrium (touchdown PCR dataset)

For the touchdown PCR dataset, analysis of alpha diversity showed that the richness of the bacterial profiles of the cervix and endometrium did not differ from those of background technical controls (Supplementary Fig. S1, Table S3). However, both the cervix and endometrium had greater bacterial profile heterogeneity than the technical controls (Supplementary Table S3).

**Figure 5:**
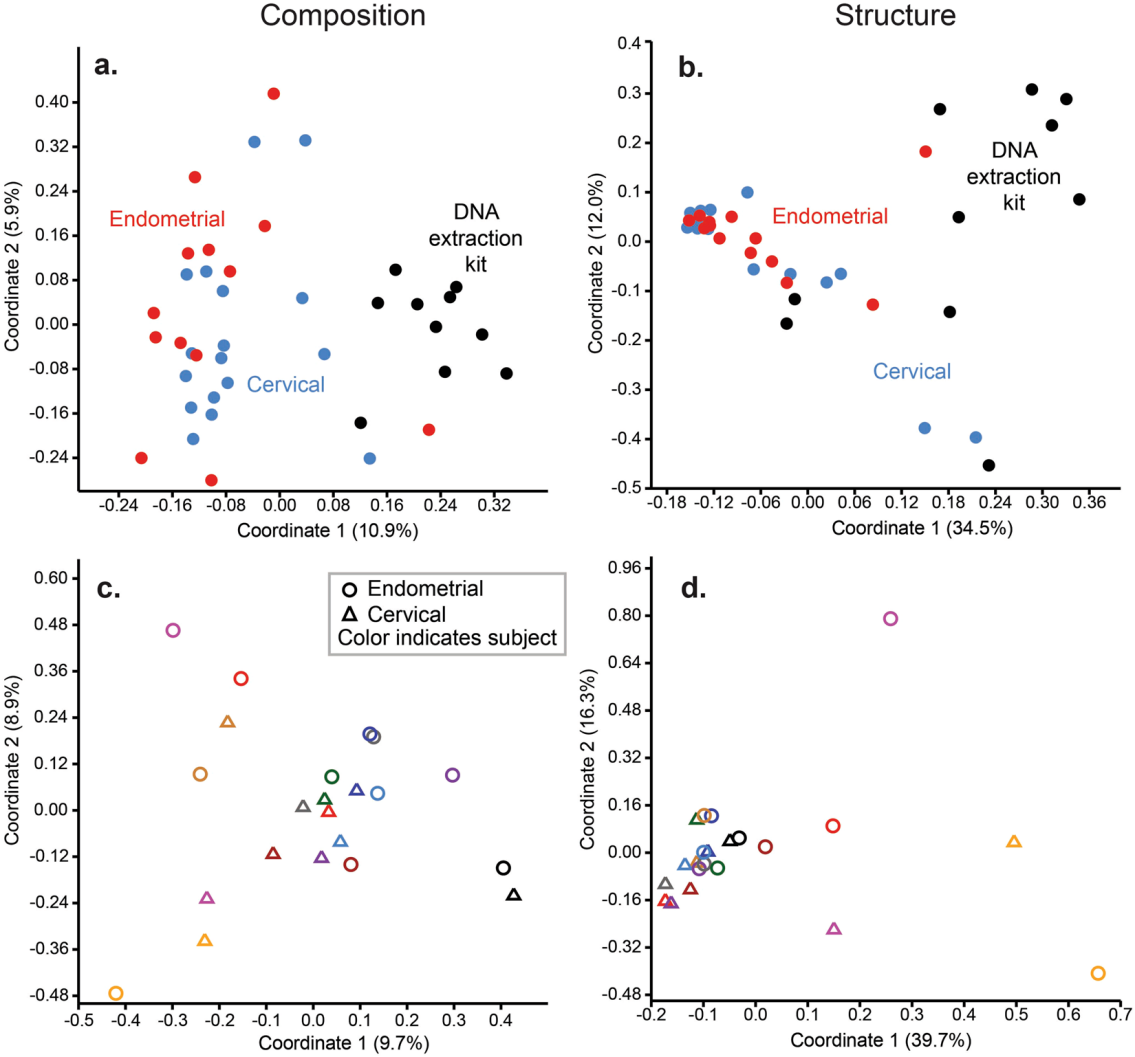
Principal Coordinates Analyses (PCoA) illustrating differences in 16S rRNA gene profiles among cervical, endometrial, and DNA extraction kit samples using a touchdown PCR approach. Profiles were generated for 16S rRNA gene community composition and structure using the Jaccard and Bray-Curtis indices, respectively. In panels c and d, the color coding of circles (endometrial) and triangles (cervical) indicates subject (i.e. patient) identity.

With respect to beta diversity, there was separation between the composition and structure of the bacterial profiles of cervical and endometrial samples and those of technical controls (Fig. 5a,b; PERMANOVA, *p* = 0.0001). However, the bacterial profiles of the cervix and endometrium did not consistently differ from each other (*p* > 0.05). Instead, subject (i.e. patient) identity explained most of the variation in bacterial profiles among the cervical and endometrial samples (Jaccard: subject, R^2^ = 0.49, *p* = 0.002, sample type R^2^ = 0.04, *p* = 0.50; Bray-Curtis: subject, R^2^ = 0.56, *p* = 0.001, sample type, R^2^ = 0.05, *p* = 0.09) (Fig. 5c,d).

**Figure 6:**
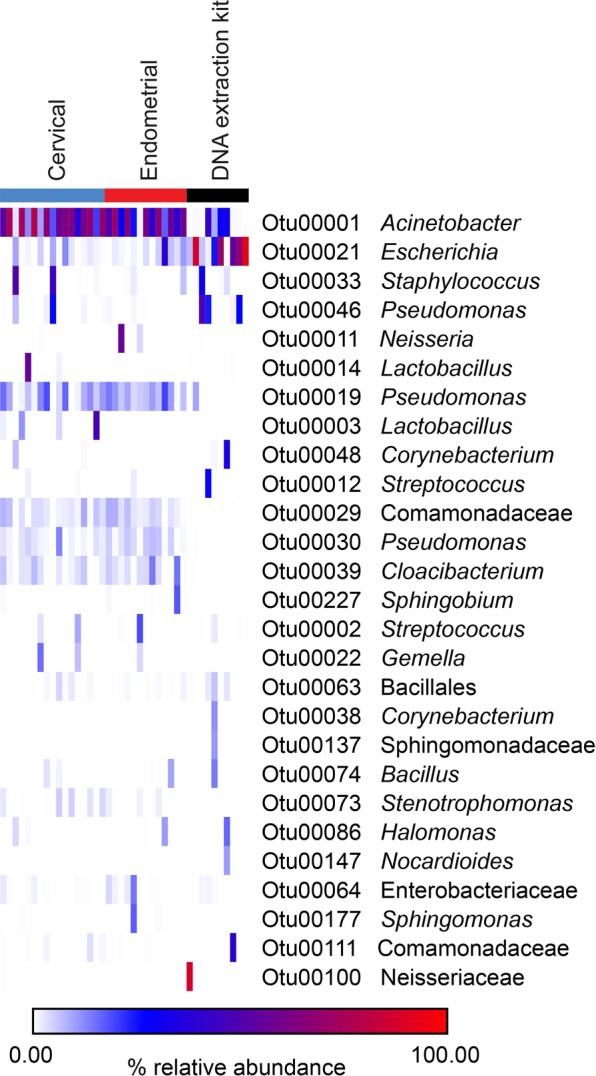
Heat map illustrating percent relative abundances of prominent operational taxonomic units (≥1% average relative abundance) among cervical, endometrial, and DNA extraction kit samples. Amplification of 16S rRNA genes was performed using a touchdown PCR approach.

Investigation of the taxonomic identities of prominent OTUs (≥1% average relative abundance) by sample type in the touchdown PCR dataset (Fig. 6) was congruent with analyses for the standard PCR dataset. Specifically, the bacterial profiles of cervical and endometrial samples were dominated by *Acinetobacter* (OTU 1), which accounted for 49.0% and 44.4% of sequences from these body sites, respectively. Other prominent taxa in the endometrium included *Pseudomonas* (OTUs 19 and 30), *Cloacibacterium* (OTU 39), Comamonadaceae (OTU 29), and *Escherichia* (OTU 21). Other prominent taxa in the cervix included *Pseudomonas* (OTUs 19 and 30), *Cloacibacterium* (OTU 39), Comamonadaceae (OTU 29), *Lactobacillus* (OTUs 3 and 14), *Escherichia* (OTU 21), and *Staphylococcus* (OTU 33). Nine different *Lactobacillus* OTUs were identified in 14/17 (82.4%) cervical samples (one to three *Lactobacillus* OTUs per sample), wherein they accounted for 42,346/436,436 (9.7%) sequences. Among endometrial samples, a maximum of one *Lactobacillus* OTU (either OTU 3, OTU 14, or OTU 25) was detected in 3/13 (23.1%) samples. These three *Lactobacillus* OTUs accounted for only 11/62,325 (0.018%) sequences among these three endometrial samples.

The most widespread and abundant genus in the profiles of background technical controls was *Escherichia* (OTU 21), with an average relative abundance of 41.3% (Fig. 6). Other prominent taxa in the control samples were *Acinetobacter* (OTU1; 11.5%), *Pseudomonas* (OTU 46; 10.4%), and *Staphylococcus* (OTU 33; 3.6%).

**Figure 7:**
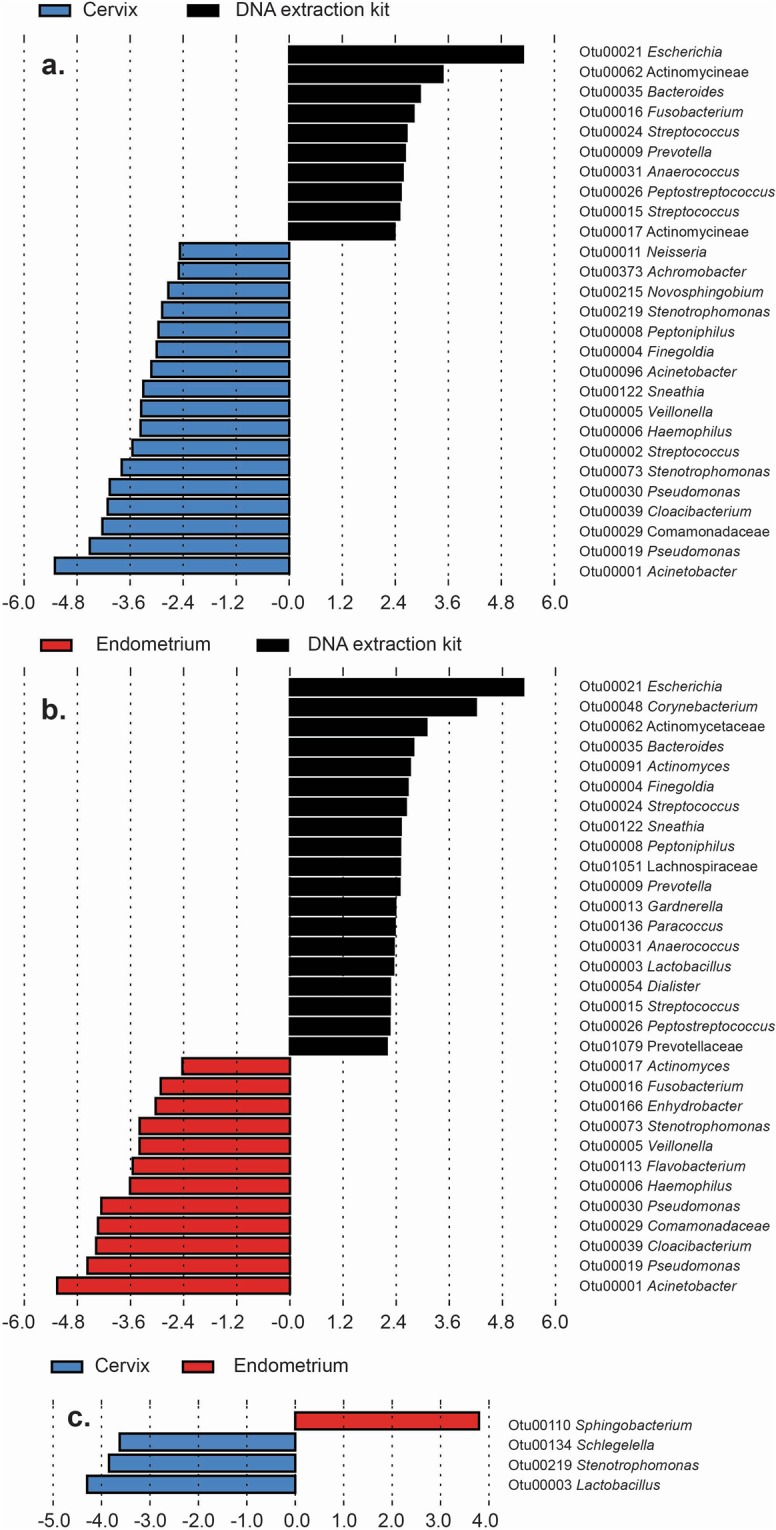
Linear discriminant analysis effect size (LEfSe) of bacterial operational taxonomic units (OTUs) with significant differential abundance in cervical, endometrial, and DNA extraction kit samples. Classification of differential OTUs between (**a**) the cervix and DNA extraction kits, (**b**) the endometrium and DNA extraction kits, and (**c**) the cervix and endometrium.

Linear discriminant analysis effect size (LEfSe) identified OTUs that were differentially abundant between the cervix, endometrium, and background technical controls (Fig. 7 and Supplementary Table S4). Twenty-seven OTUs had differential relative abundances between the cervical and control samples, and 31 OTUs had differential relative abundances between endometrial and control samples. *Acinetobacter* (OTU 1), *Pseudomonas* (OTUs 19 and 30), Comamonadaceae (OTU 29), and *Cloacibacterium* (OTU 39) were most differentially abundant between the cervical and endometrial samples and background technical controls. Four OTUs had differential relative abundances between cervical and endometrial samples: *Lactobacillus*, *Stenotrophomonas*, and *Schlegelella* (OTUs 3, 219, and 134, respectively) were more relatively abundant in the cervix than the endometrium, and *Sphingobacterium* (OTU 110) was more abundant in the endometrium than the cervix.

### Amplicon sequence variant analysis of the cervix, endometrium, and background technical controls

Amplicon sequence variants within 0–1 base pair similarity of the consensus sequences for *Acinetobacter* (OTU 1), *Pseudomonas* (OTUs 19 and 30), Comamonadaceae (OTU 29), and *Cloacibacterium* (OTU 39) were identified in cervical, endometrial, and background technical control samples (Supplementary Fig. S2). These five OTUs were prominent among cervical and endometrial samples (Fig. 6), and were indicated by LEfSe analysis as being relatively more abundant in both cervical and endometrial samples than in background technical controls (Fig. 7). In the standard PCR dataset, amplicon sequence variants differing at most by one base pair from the consensus sequences of these prominent OTUs were widespread and relatively abundant among cervical and endometrial samples, yet they were rarely identified in technical controls (Supplementary Fig. S2). In the touchdown PCR dataset, amplicon sequence variants differing at most by one base pair from the consensus sequences of these prominent OTUs were again either widespread and relatively abundant among cervical and endometrial samples, yet rarely identified in technical controls (OTUs 30, 29, and 39), or they were widespread among cervical, endometrial, and technical controls, yet present at much higher relative abundances among the cervical and endometrial samples (OTUs 1 and 19) (Supplementary Fig. S2). Most notably, the amplicon sequence variant that was identical to the consensus sequence of OTU 1 was detected in all cervical samples and in 14/15 endometrial samples, with average relative abundances of 44.9% and 32.7%, respectively (Supplementary Fig. S2). This sequence variant was detected in each of the eight control samples, but at much lower relative abundances (0.03% to 1.15%) than in the cervical and endometrial samples.

### *Lactobacillus*-specific quantitative real-time PCR of vaginal, cervical, and endometrial samples

**Figure 8:**
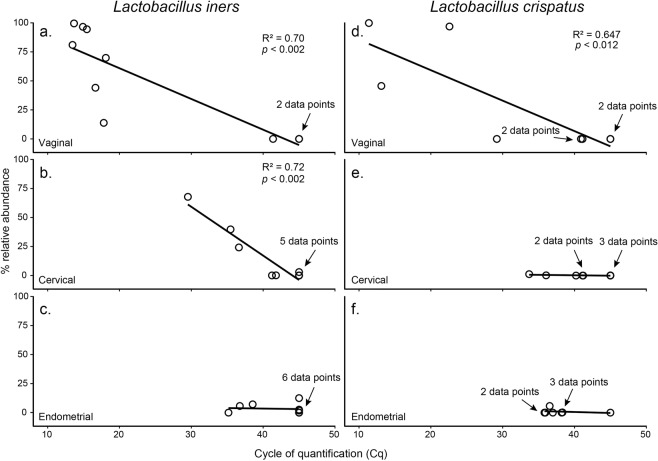
Regressions of the relative (MiSeq sequencing data) and absolute (qPCR Cq data) abundances of *Lactobacillus iners* and *L*. *crispatus* in vaginal, cervical, and endometrial samples.

Two targeted qPCR assays were performed to investigate the presence and absolute abundance of *L*. *iners* and *L*. *crispatus* in cervical and endometrial samples. Representative subjects (i.e. patients) were selected for analysis based on the relative abundances of OTU 3 (*L*. *iners*) and OTU 14 (*L*. *crispatus*) in their vaginal samples. Specifically, representative samples were selected to cover a broad range of relative abundances within the vagina for *L*. *iners* (N = 10; 0.02–99.5%) and *L*. *crispatus* (N = 8; 0.001–99.7%). Each subject’s vaginal, cervical, and endometrial samples were assayed for the absolute abundance of the respective *Lactobacillus* species. The qPCR assays demonstrated an absence or very low abundance of *Lactobacillus* in the endometrium, even when *Lactobacillus* was abundant in the vagina (Fig. 8).

For *L*. *iners*, a strong correlation between the relative (MiSeq sequence data) and absolute (qPCR Cq data) abundances was observed for vaginal **(**Fig. 8a**)** and cervical samples **(**Fig. 8b**)**, but not for endometrial samples **(**Fig. 8c**)**. The *L*. *iners* assay resulted in positive amplification (Cq < 35) for 7/10 (70%) vaginal samples, 1/10 (10%) cervical samples, and 0/10 (0%) endometrial samples. The vaginal sample with the highest relative abundance for *L*. *iners* (99.5%) in its respective MiSeq library had a Cq value of 13.7 for the *L*. *iners* qPCR assay. The three vaginal samples with the lowest relative abundances for *L*. *iners* (each < 1%) in their respective MiSeq libraries had Cq values > 35.

For *L*. *crispatus*, a strong correlation between the relative (MiSeq) and absolute (qPCR) abundances was observed only in vaginal samples **(**Fig. 8d**)**. The *L*. *crispatus* assay resulted in positive amplification for 4/8 (50%) vaginal samples, 1/8 (12.5%) cervical samples **(**Figs. 8e), and 0/8 (0%) endometrial samples **(**Fig. 8f**)**. The vaginal sample with the highest relative abundance for *L*. *crispatus* (99.7%) in its respective MiSeq library had a Cq value of 11.4 for the *L*. *crispatus* qPCR assay. The three vaginal samples with the lowest relative abundances for *L*. *crispatus* (each <0.005%) in their respective MiSeq libraries had Cq values > 35. Thus, neither *L*. *iners* nor *L*. *crispatus* was widespread or abundant among endometrial samples **(**Fig. 8c,f).

## Discussion

The principal findings of the study were: 1) 60% (15/25) of the middle endometrial samples had a bacterial load exceeding that of background DNA controls; 2) Bacterial loads in the endometrium were correlated with those in the cervix (R^2^ = 0.32, *p* = 0.003), but not with those in the vagina; 3) Among women with bacterial loads in the endometrium exceeding those of background controls, endometrial bacterial profiles were distinct from those of the controls and other body sites, except the cervix; 4) Endometrial and cervical bacterial profiles were similar when characterized using standard and touchdown PCR approaches: in both instances, they were dominated by *Acinetobacter*, *Pseudomonas*, *Cloacibacterium*, and Comamonadaceae; 5) *Lactobacillus* was dominant in vaginal bacterial profiles, and it was widespread among cervical profiles, yet it was rare in the bacterial profiles of endometrial samples using 16S rRNA gene sequencing (i.e. in 23% (3/13) of endometrial samples, accounting for 0.006% of sequence reads in these three samples, within the touchdown PCR dataset); and 6) Using species-specific qPCR, *Lactobacillus iners* and *L*. *crispatus* were detected (i.e. Cq < 35) among vaginal samples with a spectrum of *L*. *iners* and *L*. *crispatus* relative abundances (13.8% − 99.5% and 0.008% - 99.7%, respectively), yet neither species was detected by qPCR in endometrial samples.

Recent studies concluding the existence of a resident endometrial microbiota through molecular techniques have reported varying taxonomic structures for this microbiota^24–37,78^ **(**Supplementary Table S1**)**. Most studies in which endometrial samples were collected transcervically have reported that *Lactobacillus*, a genus commonly dominant in the human vagina^79–85^, is also dominant in the endometrium^25–28,33–37,78^. The exception was a study by Verstraelen *et al*.^29^, which reported that 90% of women had endometrial bacterial profiles in which three *Bacteroides* and one *Pelomonas* species were most abundant. The presence and relative abundance of *Lactobacillus* in studies in which endometrial samples were not collected transcervically has been more variable^24,30–32^.

Using multiple species-specific qPCR assays to detect prevalent vaginal bacteria in vaginal, upper endocervical, and endometrial samples from 58 women who underwent hysterectomy, Mitchell *et al*.^24^ determined that 95% of the women exhibited colonization of the endocervix and/or the endometrium by at least one bacterial species. The most commonly detected bacteria in the vagina were *Prevotella* spp. (76%), *L*. *iners* (61%), and *L*. *crispatus* (56%). *L*. *iners* (45%), *L*. *crispatus* (33%), and *Prevotella* spp. (33%) were also the most often detected bacteria among the endocervical/endometrial samples (endocervical and endometrial samples were considered collectively as being representative of the upper genital tract)^24^. Chen *et al*.^31^ recently used 16S rRNA gene sequencing to analyze the bacterial profiles of the lower and upper reproductive tracts of 110 women who underwent hysterectomy, and reported that the relative abundances of *Lactobacillus* decreased from the lower reproductive tract (median abundance of 99.9% in the vagina) to the upper reproductive tract (median abundance of 1.7% in the fallopian tubes). In the endometrium specifically, *Lactobacillus* remained relatively abundant (30.6%), but high relative abundances of *Acinetobacter* (9.1%), *Pseudomonas* (9.1%), *Vagococcus* (7.3%), *Sphingobium* (5.0%), and Comamonadaceae (4.9%) were also observed. Miles *et al*.^32^ used 16S sequencing to investigate the presence of a microbiota in the reproductive tract of 10 women who underwent a total hysterectomy with bilateral salpingo-oopherectomy, and found that *Lactobacillus* was present in approximately half of the vaginal, cervical, endometrial, and myometrial samples at varying abundances. *Acinetobacter* and *Corynebacterium*, while not present in vaginal samples, were detected among cervical, endometrial, and myometrial samples. Lastly, Walther-António *et al*.^30^ used 16S sequencing to characterize the endometrial bacterial profiles of 10 women who underwent hysterectomy for non-malignant uterine conditions, and reported that *Lactobacillus* was infrequently detected, and then at only low relative abundances, within the endometrium. The most prominent bacteria in the endometrium were instead *Shigella* and *Barnesiella*. Therefore, although *Lactobacillus* has been reported as being widespread and dominant in endometrial samples collected transcervically, its distribution and abundance among endometrial samples that were not collected transcervically has been more variable.

In the current study, samples were obtained from the mid-endometrium after hysterectomy, and the endometrial microbiota was not dominated by *Lactobacillus*. Instead, endometrial bacterial profiles were largely dominated by *Acinetobacter*, *Pseudomonas*, Comamonadaceae, and *Cloacibacterium*. Each of these taxa has previously been detected in the human endometrium using molecular techniques^25,26,28–33,35,36,78^ **(**Supplementary Table S4**)**. In general, our findings are consistent with those of Chen *et al*.^31^ and Miles *et al*.^32^ in that we found decreasing levels of *Lactobacillus* from the vagina and the cervix to the endometrium. The rarity of *Lactobacillus* in the mid-endometrium in the current study suggests that the vaginal microbiota may not be a persistent source of microbes for the endometrial microbiota. This is further supported by our finding that bacterial load in the vagina did not predict bacterial load in either the cervix or the endometrium. Instead, it was bacterial load in the cervix that was correlated with bacterial load in the endometrium, and, while the bacterial profiles of the cervix and endometrium were similar, they were both distinct from the bacterial profile of the vagina.

Despite overall similarities in the bacterial profiles of the endometrium and the cervix, some differences were still evident. Most notably, LEfSe analyses indicated that *Lactobacillus* was more widespread and relatively abundant among cervical than endometrial samples. This finding is consistent with prior molecular studies that reported the presence of *Lactobacillus* in the cervix^31,32,86,87^. In the current study, the average relative abundance of *Lactobacillus* in the cervix was 19.24%. Other studies have reported a range of relative abundances of *Lactobacillus* in the cervix from 10–99%^31,32,86,87^. It is unclear why, in the current study, *Lactobacillus* was dominant in the vagina, common in the cervix, and yet very rare in the endometrium. Ascension of vaginal bacteria through the cervix has been suggested as a likely source of bacterial transmission to the endometrium^24,88–91^. For example, in an experimental study, it was shown that labeled spermatozoa-size particles (i.e. macroaggregates of human serum albumin) can translocate from the vagina to the uterus through the cervical canal within minutes in non-pregnant women^92^. Thus, the uterine peristaltic pump that aids in sperm transport from the cervical canal to the endometrium^93^ might also play a role in seeding the endometrium with bacteria. However, the powerful antimicrobial activity of the cervix and cervical secretions^94–111^ may act as an effective barrier separating the endometrium from the microbe-rich vagina. The findings of the current study suggest that the cervix may act as an effective barrier against the ascension of vaginal microbes into the endometrium. An alternative explanation is that vaginal microbes, including *Lactobacillus*, do ascend through the cervix but that their absolute and relative abundances rapidly decrease from the endocervix to the lower, middle, and upper endometrium. This may explain why *Lactobacillus* was more often detected in the endocervix/endometrium (i.e. upper genital tract) among women undergoing hysterectomies in the study performed by Mitchell *et al*.^24^ than in the current study in which the mid-endometrium was targeted. The extent to which the vaginal and cervical microbiotas are a persistent and sustained source of bacterial populations for an endometrial microbiota, and explanations for why *Lactobacillus* immigration and colonization appears inhibited, remain to be elucidated.

The current study suggests that, if there is an endometrial microbiota, *Acinetobacte*r, *Pseudomonas*, Comamonadaceae, and *Cloacibacterium* are its principal members. Chen *et al*.^31^ previously reported that *Acinetobacter*, *Pseudomonas*, and Comamonadaceae were among the most relatively abundant bacteria within the endometrium, pouch of Douglas, and fallopian tubes, and Miles *et al*.^32^ detected *Acinetobacter* in the cervix, endometrium, and the myometrium. Notably, *Acinetobacter* has been previously cultured from both the cervix^112^ and the endometrium^17,113,114^. *Pseudomonas*^17,114^ has been cultured from the endometrium as well.

While the exact taxonomy (i.e. species) of the prominent *Acinetobacter* OTU detected in the endometrium is unknown, the sequence analysis we conducted using BLAST showed that its consensus sequence was identical to sequences of eight *Acinetobacter* type strains that have been previously isolated from clinical samples^115–121^. Sequence analysis of the two prominent *Pseudomonas* OTUs in endometrial samples in this study showed that one belongs to the *P*. *aeruginosa* group and the other to the *P*. *putida* group^77^. The consensus sequence of the prominent *Cloacibacterium* OTU is identical to a strain of *Cloacibacterium normanense*, a species that was detected in a tissue sample of a patient with spondylodiscitis^122^. Lastly, the consensus sequence of the prominent Comamonadaceae OTU was identical to a strain of *Comamonas kerstersii*, a documented opportunistic pathogen that has been isolated from clinical samples^123–127^. Notably, these endometrial and cervical OTUs were also detected at low relative abundances in vaginal samples in this study. Similarly, in prior studies, *Acinetobacter*^80,128^, *Pseudomonas*^80,128^, and *Cloacibacterium*^128^ have been detected in vaginal microbial communities, even when *Lactobacillus* was dominant. These taxa may therefore be common members of both the lower and upper reproductive tract. Whether the molecular signals in the current study represent a viable and residential endometrial microbiota, and, if so, whether *Acinetobacter*, *Pseudomonas*, Comamonadaceae, and *Cloacibacterium* play a role in women’s reproductive health, warrants further investigation.

It is important to note that *Acinetobacter*, *Pseudomonas*, Comamonadaceae, and *Cloacibacterium* have each been implicated as a contaminant in at least one prior molecular survey study **(**Supplementary Table S4**)**. However, in the current study, the distributions and abundances of these bacterial taxa among biological samples and technical controls were not consistent with their being background DNA contaminants. For example, among the 10 blank DNA extraction kit controls we sequenced, Comamonadaceae was represented by only a single sequence read using standard PCR and four total sequence reads using touchdown PCR. Additionally, among endometrial samples, the relative abundances of Comamonadaceae were not negatively correlated with overall 16S rRNA gene abundances (i.e. bacterial load, as determined by qPCR). A negative correlation between bacterial load and OTU relative abundance is a pattern predictive of, and used by others to identify, background DNA contaminants among low biomass samples^40,129^. Similarly, the relative abundances of the two *Pseudomonas* OTUs and *Cloacibacterium* were also not negatively correlated with 16S rRNA gene abundances among the endometrial samples. Each of these taxa were detected in at most one-half of the background technical controls. Although *Acinetobacter* was identified among the bacterial profiles of technical controls, the relative abundances of OTU 1 (*Acinetobacter*) were positively correlated with overall 16S rRNA gene abundances (i.e., bacterial load) among endometrial samples (R^2^ = 0.32, *p* = 0.003). This pattern is opposite that predicted for a background DNA contaminant. Furthermore, sequence variant analysis identified diverse *Acinetobacter*, *Pseudomonas*, Comamonadaceae, and *Cloacibacterium* sequence variants that were widespread among endometrial and cervical samples and yet were not present in background technical control samples. Together, these findings suggest that molecular signals of *Acinetobacter*, *Pseudomonas*, Comamonadaceae, and *Cloacibacterium* identified in endometrium samples are not consistent with background DNA contamination. Instead, they are suggestive of a potential resident microbiota in the endometrium whose existence must be verified through microscopy and culture.

The current study has four principal strengths. First, we limited our investigation to samples that were obtained following hysterectomy, thereby reducing the likelihood of contamination from the lower urogenital tract^2,10,22^. Second, we used multiple, complementary modes of inquiry: 16S rRNA gene sequencing, universal 16S rRNA gene qPCR, and *Lactobacillus*-specific targeted 16S rRNA gene qPCR. Third, we incorporated technical controls for potential background DNA contamination. Fourth, we restricted our bacterial profile analyses to samples that had both a substantive bacterial load based on the results of qPCR (i.e. Cq < 35) and that had thorough sample coverage (i.e. >98%) based on 16S rRNA gene sequencing.

The current study has four primary limitations. First, all women included in the study underwent hysterectomy for gynecological disease, as endometrial biopsies from healthy individuals are rarely available. While most of the women had uterine fibroids, a condition not commonly associated with infection^130^, it is unclear whether the bacterial communities detected represent a normal endometrial microbiota. Second, the study was limited to the middle endometrium. Further investigation and potential delineation of a lower, middle, and upper endometrial microbiota is required. Third, swabs were not included as extraction controls. Nevertheless, there were no negative correlations between the abundances of any of the candidate endometrial microbes and sample DNA concentrations as assessed through 16S rRNA gene qPCR, a pattern illustrative of DNA contaminants. Fourth, this was a molecular microbiology study without culture and imaging components to demonstrate viability and localization of microbial communities associated with endometrial tissues^129,131^.

In conclusion, using complementary modes of inquiry, we detected a bacterial signature within the middle endometrium of 60% of women who underwent hysterectomy. The endometrial bacterial profiles were distinct from those of background technical controls and other body sites, except the cervix. In contrast to the vagina and cervix, *Lactobacillus* was rarely detected in the endometrium suggesting that the vaginal microbiota is not a persistent source of microbes for an endometrial microbiota. This is further supported by the finding that bacterial load in the vagina did not predict bacterial load in either the cervix or endometrium. Instead it was bacterial load in the cervix that was correlated with bacterial load in the endometrium, suggesting that, if an endometrial microbiota exists, the cervical microbiota may be a persistent source of colonizing microbes, or vice versa. The results of this study show that molecular signals from *Acinetobacter*, *Pseudomonas*, Comamonadaceae, and *Cloacibacterium*, but not *Lactobacillus*, are relatively abundant within the human mid-endometrium. The existence and viability of an endometrial microbiota must be confirmed through microscopy and culture^129,131^, and longitudinal studies of lower, middle, and upper endometrial microbiotas are necessary. Nevertheless, the extent to which the detected molecular signals of a potential endometrial microbiota contribute to female reproductive health and disease warrants further consideration.

## Supplementary information

Supplementary Methods

Supplementary Data

## Data Availability

The MiSeq. 16S rRNA gene sequence data generated during the current study have been deposited on the NCBI Sequence Read Archive (BioProject ID PRJNA543861). The raw operational taxonomic unit (OTU) data from this study are provided as Supplementary Data.

## References

[1] Butler B (1958). Value of endometrial cultures in sterility investigation. Fertil Steril.

[2] Bollinger CC (1964). Bacterial flora of the nonpregnant uterus: a new culture technic. Obstet Gynecol.

[3] Mishell DR, Bell JH, Good RG, Moyer DL (1966). The intrauterine device: a bacteriologic study of the endometrial cavity. Am J Obstet Gynecol.

[4] Ansbacher R, Boyson WA, Morris JA (1967). Sterility of the uterine cavity. Am J Obstet Gynecol.

[5] Spore WW, Moskal PA, Nakamura RM, Mishell DR (1970). Bacteriology of postpartum oviducts and endometrium. Am J Obstet Gynecol.

[6] Grossman JH, Adams RLHWJ, Andriole VT (1978). Endometrial and vaginal cuff bacteria recovered at elective hysterectomy during a trial of antibiotic prophylaxis. Am J Obstet Gynecol.

[7] Pezzlo MT, Hesser JW, Morgan T, Valter PJ, Thrupp LD (1979). Improved laboratory efficiency and diagnostic accuracy with new double-lumen-protected swab for for endometrial specimens. J Clin Microbiol.

[8] Cooperman NR, Kasim M, Rajashekaraiah KR (1980). Clinical significance of amniotic fluid, amniotic membranes, and endometrial biopsy cultures at the time of cesarean section. Am J Obstet Gynecol.

[9] Sparks RA, Purrier BG, Watt PJ, Elstein M (1981). Bacteriological colonisation of uterine cavity: role of tailed intrauterine contraceptive device. Br Med J (Clin Res Ed).

[10] Duff P, Gibbs RS, Blanco JD, St PC (1983). Endometrial culture techniques in puerperal patients. Obstet Gynecol.

[11] Duff P, Gibbs RS, St Clair PJ, Weinberg LC (1984). Correlation of laboratory and clinical criteria in the prediction of postcesarean endomyometritis. Obstet Gynecol.

[12] Awadalla SG, Perkins RP, Mercer LJ (1986). Significance of endometrial cultures performed at cesarean section. Obstet Gynecol.

[13] Eschenbach DA, Rosene K, Tompkins LS, Watkins H, Gravett MG (1986). Endometrial cultures obtained by a triple-lumen method from afebrile and febrile postpartum women. J Infect Dis.

[14] Nelson LH, Nichols SB (1986). Effectiveness of the Isaacs cell sampler for endometrial cultures. J Reprod Med.

[15] Teisala K (1987). Endometrial microbial flora of hysterectomy specimens. Eur J Obstet Gynecol Reprod Biol.

[16] Møller BR, Kristiansen FV, Thorsen P, Frost L, Mogensen SC (1995). Sterility of the uterine cavity. Acta Obstet Gynecol Scand.

[17] Drbohlav P (1998). The effect of endometrial infection on embryo implantation in the IVF and ET program. Ceska Gynekol.

[18] Cicinelli E (2015). Prevalence of chronic endometritis in repeated unexplained implantation failure and the IVF success rate after antibiotic therapy. Hum Reprod.

[19] Naessens A, Foulon W, Cammu H, Goossens A, Lauwers S (1987). Epidemiology and pathogenesis of ureaplasma urealyticum in spontaneous abortion and early preterm labor. Acta Obstet Gynecol Scand.

[20] Cicinelli E (2014). Chronic endometritis due to common bacteria is prevalent in women with recurrent miscarriage as confirmed by improved pregnancy outcome after antibiotic treatment. Reprod Sci.

[21] Gravett MG, Hitti J, Hess DL, Eschenbach DA (2000). Intrauterine infection and preterm delivery: evidence for activation of the fetal hypothalamic-pituitary-adrenal axis. Am J Obstet Gynecol.

[22] Romero R, Espinoza J, Mazor M (2004). Can endometrial infection/inflammation explain implantation failure, spontaneous abortion, and preterm birth after *in vitro* fertilization?. Fertil Steril.

[23] Marchesi JR, Ravel J (2015). The vocabulary of microbiome research: a proposal. Microbiome.

[24] Mitchell CM (2015). Colonization of the upper genital tract by vaginal bacterial species in nonpregnant women. Am J Obstet Gynecol.

[25] Fang RL (2016). Barcoded sequencing reveals diverse intrauterine microbiomes in patients suffering with endometrial polyps. Am J Transl Res.

[26] Franasiak JM (2016). Endometrial microbiome at the time of embryo transfer: next-generation sequencing of the 16S ribosomal subunit. Journal of Assisted Reproduction and Genetics.

[27] Khan KN (2016). Molecular detection of intrauterine microbial colonization in women with endometriosis. Eur J Obstet Gynecol Reprod Biol.

[28] Moreno I (2016). Evidence that the endometrial microbiota has an effect on implantation success or failure. Am J Obstet Gynecol.

[29] Verstraelen H (2016). Characterisation of the human uterine microbiome in non-pregnant women through deep sequencing of the V1-2 region of the 16S rRNA gene. PeerJ.

[30] Walther-Antonio MR (2016). Potential contribution of the uterine microbiome in the development of endometrial cancer. Genome Med.

[31] Chen C (2017). The microbiota continuum along the female reproductive tract and its relation to uterine-related diseases. Nat Commun.

[32] Miles SM, Hardy BL, Merrell DS (2017). Investigation of the microbiota of the reproductive tract in women undergoing a total hysterectomy and bilateral salpingo-oopherectomy. Fertil Steril.

[33] Tao X (2017). Characterizing the endometrial microbiome by analyzing the ultra-low bacteria from embryo transfer catheter tips in IVF cycles: Next generation sequencing (NGS) analysis of the 16S ribosomal gene. Human Microbiome Journal.

[34] Kyono K, Hashimoto T, Nagai Y, Sakuraba Y (2018). Analysis of endometrial microbiota by 16S ribosomal RNA gene sequencing among infertile patients: a single-center pilot study. Reprod Med Biol.

[35] Liu Y (2018). Systematic Comparison of Bacterial Colonization of Endometrial Tissue and Fluid Samples in Recurrent Miscarriage Patients: Implications for Future Endometrial Microbiome Studies. Clin Chem.

[36] Pelzer, E. S., Willner, D., Buttini, M. & Huygens, F. A role for the endometrial microbiome in dysfunctional menstrual bleeding. *Antonie Van Leeuwenhoek* **111**, 933–943 (2018). 10.1007/s10482-017-0992-6 29299770

[37] Wee BA (2018). A retrospective pilot study to determine whether the reproductive tract microbiota differs between women with a history of infertility and fertile women. Aust N Z J Obstet Gynaecol.

[38] Giudice LC (2016). Challenging dogma: the endometrium has a microbiome with functional consequences!. Am J Obstet Gynecol.

[39] Baker JM, Chase DM, Herbst-Kralovetz MM (2018). Uterine Microbiota: Residents, Tourists, or Invaders?. Front Immunol.

[40] Salter SJ (2014). Reagent and laboratory contamination can critically impact sequence-based microbiome analyses. BMC Biol.

[41] Weiss S (2014). Tracking down the sources of experimental contamination in microbiome studies. Genome Biol.

[42] Glassing A, Dowd SE, Galandiuk S, Davis B, Chiodini RJ (2016). Inherent bacterial DNA contamination of extraction and sequencing reagents may affect interpretation of microbiota in low bacterial biomass samples. Gut Pathog.

[43] Lauder AP (2016). Comparison of placenta samples with contamination controls does not provide evidence for a distinct placenta microbiota. Microbiome.

[44] Kim D (2017). Optimizing methods and dodging pitfalls in microbiome research. Microbiome.

[45] Dickson RP (2014). Changes in the Lung Microbiome following Lung Transplantation Include the Emergence of Two Distinct Pseudomonas Species with Distinct Clinical Associations. Plos One.

[46] Frank JA (2008). Critical evaluation of two primers commonly used for amplification of bacterial 16S rRNA genes. Applied and Environmental Microbiology.

[47] Fredricks DN, Fiedler TL, Thomas KK, Mitchell CM, Marrazzo JM (2009). Changes in vaginal bacterial concentrations with intravaginal metronidazole therapy for bacterial vaginosis as assessed by quantitative PCR. J Clin Microbiol.

[48] Srinivasan S (2010). Temporal variability of human vaginal bacteria and relationship with bacterial vaginosis. Plos One.

[49] Quinn, G. P. & Keough, M. J. Experimental design and data analysis for biologists. (Cambridge University Press, 2002).

[50] Gotelli, N. J. & Ellison, A. M. A primer of Ecological Statistics. (Sinauer Associates Inc. Sunderland. MA, 2004).

[51] Hammer, Ø. PAST 3 (Paleontological Statistics) version. 3.11. Home page at, http://folk. uio. no/ohammer/past (2015).

[52] Yang B, Wang Y, Qian PY (2016). Sensitivity and correlation of hypervariable regions in 16S rRNA genes in phylogenetic analysis. BMC Bioinformatics.

[53] Leung MH, Wilkins D, Lee PK (2015). Insights into the pan-microbiome: skin microbial communities of Chinese individuals differ from other racial groups. Sci Rep.

[54] Fan X, Peters BA, Min D, Ahn J, Hayes RB (2018). Comparison of the oral microbiome in mouthwash and whole saliva samples. Plos One.

[55] Segal LN (2016). Enrichment of the lung microbiome with oral taxa is associated with lung inflammation of a Th17 phenotype. Nat Microbiol.

[56] Halfvarson J (2017). Dynamics of the human gut microbiome in inflammatory bowel disease. Nat Microbiol.

[57] Jasarevic E, Howard CD, Misic AM, Beiting DP, Bale TL (2017). Stress during pregnancy alters temporal and spatial dynamics of the maternal and offspring microbiome in a sex-specific manner. Sci Rep.

[58] Hilt EE (2014). Urine is not sterile: use of enhanced urine culture techniques to detect resident bacterial flora in the adult female bladder. J Clin Microbiol.

[59] Parnell LA (2017). Microbial communities in placentas from term normal pregnancy exhibit spatially variable profiles. Sci Rep.

[60] Kozich JJ, Westcott SL, Baxter NT, Highlander SK, Schloss PD (2013). Development of a dual-index sequencing strategy and curation pipeline for analyzing amplicon sequence data on the MiSeq Illumina sequencing platform. Appl Environ Microbiol.

[61] Don RH, Cox PT, Wainwright BJ, Baker K, Mattick JS (1991). ‘Touchdown’ PCR to circumvent spurious priming during gene amplification. Nucleic Acids Res.

[62] Bassis CM (2015). Analysis of the upper respiratory tract microbiotas as the source of the lung and gastric microbiotas in healthy individuals. MBio.

[63] Dickson RP (2016). Enrichment of the lung microbiome with gut bacteria in sepsis and the acute respiratory distress syndrome. Nat Microbiol.

[64] Singer BH (2018). Bacterial Dissemination to the Brain in Sepsis. Am J Respir Crit Care Med.

[65] Schloss PD (2009). Introducing mothur: open-source, platform-independent, community-supported software for describing and comparing microbial communities. Appl Environ Microbiol.

[66] Pruesse E (2007). SILVA: a comprehensive online resource for quality checked and aligned ribosomal RNA sequence data compatible with ARB. Nucleic Acids Res.

[67] Quast C (2013). The SILVA ribosomal RNA gene database project: improved data processing and web-based tools. Nucleic Acids Res.

[68] Rognes T, Flouri T, Nichols B, Quince C, Mahe F (2016). VSEARCH: a versatile open source tool for metagenomics. PeerJ.

[69] Altschul SF, Gish W, Miller W, Myers EW, Lipman DJ (1990). Basic local alignment search tool. J Mol Biol.

[70] RCore Team, R. R: a language and environment for statistical computing. Vienna: R Foundation for Statistical Computing, 2016 (2016).

[71] Bates, D., Maechler, M., Bolker, B. & Walker, S. lme4: Linear mixed-effects models using Eigen and S4. R package version 1.1–9, http://CRAN. R-project. org/package = lme4 (2015).

[72] Hothorn T, Bretz F, Westfall P (2008). Simultaneous inference in general parametric models. Biometrical journal.

[73] Anderson MJ (2001). A new method for non‐parametric multivariate analysis of variance. Austral ecology.

[74] Oksanen J (2017). Vegan: Community Ecology Package. R package version.

[75] Segata N (2011). Metagenomic biomarker discovery and explanation. Genome Biol.

[76] Callahan BJ (2016). DADA2: High-resolution sample inference from Illumina amplicon data. Nat Methods.

[77] Anzai Y, Kim H, Park JY, Wakabayashi H, Oyaizu H (2000). Phylogenetic affiliation of the pseudomonads based on 16S rRNA sequence. Int J Syst Evol Microbiol.

[78] Moreno I (2018). The diagnosis of chronic endometritis in infertile asymptomatic women: a comparative study of histology, microbial cultures, hysteroscopy, and molecular microbiology. Am J Obstet Gynecol.

[79] Lamont RF (2011). The vaginal microbiome: new information about genital tract flora using molecular based techniques. Brit J Obstet Gynaec.

[80] Ravel J (2011). Vaginal microbiome of reproductive-age women. Proc Natl Acad Sci USA.

[81] Aagaard K (2012). A metagenomic approach to characterization of the vaginal microbiome signature in pregnancy. Plos One.

[82] Petrova MI, Lievens E, Malik S, Imholz N, Lebeer S (2015). Lactobacillus species as biomarkers and agents that can promote various aspects of vaginal health. Front Physiol.

[83] Petrova MI, Reid G, Vaneechoutte M, Lebeer S (2017). Lactobacillus iners: Friend or Foe?. Trends Microbiol.

[84] Romero R (2014). The vaginal microbiota of pregnant women who subsequently have spontaneous preterm labor and delivery and those with a normal delivery at term. Microbiome.

[85] Greenbaum, S., Greenbaum, G., Moran-Gilad, J. & Weintruab, A. Y. Ecological dynamics of the vaginal microbiome in relation to health and disease. *Am J Obstet Gynecol* **220**, 324–335 (2018). 10.1016/j.ajog.2018.11.1089 30447213

[86] Smith BC (2012). The cervical microbiome over 7 years and a comparison of methodologies for its characterization. Plos One.

[87] Smith BC (2016). Distinct Ecological Niche of Anal, Oral, and Cervical Mucosal Microbiomes in Adolescent Women. Yale J Biol Med.

[88] Gomez CI, Stenback WA, James AN, Criswell BS, Williams RP (1979). Attachment of Neisseria gonorrhoeae to human sperm. Microscopical study of trypsin and iron. Br J Vener Dis.

[89] Wolner-Hanssen P, Mardh PA (1984). *In vitro* tests of the adherence of Chlamydia trachomatis to human spermatozoa. Fertil Steril.

[90] Svenstrup HF, Fedder J, Abraham-Peskir J, Birkelund S, Christiansen G (2003). Mycoplasma genitalium attaches to human spermatozoa. Hum Reprod.

[91] Heinonen PK (1985). Anatomic sites of upper genital tract infection. Obstet Gynecol.

[92] Zervomanolakis I (2007). Physiology of upward transport in the human female genital tract. Ann N Y Acad Sci.

[93] Kunz G (1997). The uterine peristaltic pump. Normal and impeded sperm transport within the female genital tract. Adv Exp Med Biol.

[94] Schumacher G, Kim M, Hosseinian A, Dupon C (1977). Immunoglobulins, proteinase inhibitors, albumin, and lysozyme in human cervical mucus: I. Communication: Hormonal profiles and cervical mucus changes—Methods and results. American Journal of Obstetrics & Gynecology.

[95] Hafez, E. S. E. *Human reproduction: conception and contraception*. (HarperCollins Publishers, 1980).

[96] Roncalli M, Sideri M, Gie P, Servida E (1988). Immunophenotypic analysis of the transformation zone of human cervix. Laboratory investigation; a journal of technical methods and pathology.

[97] Svinarich DM, Wolf NA, Gomez R, Gonik B, Romero R (1997). Detection of human defensin 5 in reproductive tissues. Am J Obstet Gynecol.

[98] Quayle AJ (1998). Gene expression, immunolocalization, and secretion of human defensin-5 in human female reproductive tract. Am J Pathol.

[99] Valore EV (1998). Human beta-defensin-1: an antimicrobial peptide of urogenital tissues. The Journal of clinical investigation.

[100] Cohen, M. Genitourinary mucosal defenses in *Sexually transmitted diseases* (ed. Holmes, K. K.) 173–190 (McGraw Hill, 1999).

[101] Fichorova RN, Anderson DJ (1999). Differential expression of immunobiological mediators by immortalized human cervical and vaginal epithelial cells. Biol Reprod.

[102] Eggert-Kruse W, Botz I, Pohl S, Rohr G, Strowitzki T (2000). Antimicrobial activity of human cervical mucus. Human reproduction.

[103] Fichorova RN, Desai PJ, Gibson FC, Genco CA (2001). Distinct proinflammatory host responses to Neisseria gonorrhoeae infection in immortalized human cervical and vaginal epithelial cells. Infect Immun.

[104] Hein M, Valore EV, Helmig RB, Uldbjerg N, Ganz T (2002). Antimicrobial factors in the cervical mucus plug. Am J Obstet Gynecol.

[105] Quayle AJ (2002). The innate and early immune response to pathogen challenge in the female genital tract and the pivotal role of epithelial cells. J Reprod Immunol.

[106] Pudney J, Quayle AJ, Anderson DJ (2005). Immunological microenvironments in the human vagina and cervix: mediators of cellular immunity are concentrated in the cervical transformation zone. Biol Reprod.

[107] Hansen LK (2014). The cervical mucus plug inhibits, but does not block, the passage of ascending bacteria from the vagina during pregnancy. Acta Obstet Gynecol Scand.

[108] Wira CR, Fahey JV, Rodriguez-Garcia M, Shen Z, Patel MV (2014). Regulation of mucosal immunity in the female reproductive tract: the role of sex hormones in immune protection against sexually transmitted pathogens. Am J Reprod Immunol.

[109] Lee SK, Kim CJ, Kim DJ, Kang JH (2015). Immune cells in the female reproductive tract. Immune Netw.

[110] Vornhagen J (2018). Human Cervical Mucus Plugs Exhibit Insufficiencies in Antimicrobial Activity Towards Group B Streptococcus. J Infect Dis.

[111] Elovitz MA (2019). Cervicovaginal microbiota and local immune response modulate the risk of spontaneous preterm delivery. Nat Commun.

[112] Panda PS, Kashyap B, Prasad S (2016). Microbiological profile of cervix of females attending *in-vitro* fertilization clinic of a tertiary care hospital, North India. Journal of Reproductive Health and Medicine.

[113] Egbase PE, Udo EE, Al-Sharhan M, Grudzinskas JG (1999). Prophylactic antibiotics and endocervical microbial inoculation of the endometrium at embryo transfer. Lancet.

[114] Devi CA, Ranjani A, Dhanasekaran D, Thajuddin N, Ramanidevi T (2013). Surveillance of multidrug resistant bacteria pathogens from female infertility cases. African Journal of Biotechnology.

[115] Bouvet PJ, Jeanjean S (1989). Delineation of new proteolytic genomic species in the genus Acinetobacter. Res Microbiol.

[116] Dijkshoorn L, Van Harsselaar B, Tjernberg I, Bouvet PJ, Vaneechoutte M (1998). Evaluation of amplified ribosomal DNA restriction analysis for identification of Acinetobacter genomic species. Syst Appl Microbiol.

[117] Karah N (2011). Species identification and molecular characterization of Acinetobacter spp. blood culture isolates from Norway. J Antimicrob Chemother.

[118] Nemec A, Dijkshoorn L, Jezek P (2000). Recognition of two novel phenons of the genus Acinetobacter among non-glucose-acidifying isolates from human specimens. J Clin Microbiol.

[119] Nemec A (2016). Taxonomy of haemolytic and/or proteolytic strains of the genus Acinetobacter with the proposal of Acinetobacter courvalinii sp. nov. (genomic species 14 sensu Bouvet & Jeanjean), Acinetobacter dispersus sp. nov. (genomic species 17), Acinetobacter modestus sp. nov., Acinetobacter proteolyticus sp. nov. and Acinetobacter vivianii sp. nov. Int J Syst Evol Microbiol.

[120] Turton JF, Shah J, Ozongwu C, Pike R (2010). Incidence of Acinetobacter species other than A. baumannii among clinical isolates of Acinetobacter: evidence for emerging species. J Clin Microbiol.

[121] Vaneechoutte M (1995). Identification of Acinetobacter genomic species by amplified ribosomal DNA restriction analysis. J Clin Microbiol.

[122] Stavnsbjerg C, Frimodt-Moller N, Moser C, Bjarnsholt T (2017). Comparison of two commercial broad-range PCR and sequencing assays for identification of bacteria in culture-negative clinical samples. BMC Infect Dis.

[123] Almuzara M (2017). Unusual presentations of Comamonas kerstersii infection. New Microbes New Infect.

[124] Almuzara MN (2013). Intra-abdominal infections due to Comamonas kerstersii. J Clin Microbiol.

[125] Biswas JS, Fitchett J, O’Hara G (2014). Comamonas kerstersii and the perforated appendix. J Clin Microbiol.

[126] Opota O (2014). Bacteremia caused by Comamonas kerstersii in a patient with diverticulosis. J Clin Microbiol.

[127] Zhou YH, Ma HX, Dong ZY, Shen MH (2018). Comamonas kerstersii bacteremia in a patient with acute perforated appendicitis: A rare case report. Medicine (Baltimore).

[128] Yeoman CJ (2013). A multi-omic systems-based approach reveals metabolic markers of bacterial vaginosis and insight into the disease. Plos One.

[129] de Goffau MC (2018). Recognizing the reagent microbiome. Nat Microbiol.

[130] Laughlin SK, Schroeder JC, Baird DD (2010). New directions in the epidemiology of uterine fibroids. Semin Reprod Med.

[131] Theis KR (2019). Does the human placenta delivered at term have a microbiota? Results of cultivation, quantitative real-time PCR, 16S rRNA gene sequencing, and metagenomics. Am J Obstet Gynecol.

