# Supplementary material for: Does the endometrial cavity have a molecular microbial signature?
Source: Sci Rep. 2019 Jul 9;9:9905. doi: 10.1038/s41598-019-46173-0 (PMC6616349; doi:10.1038/s41598-019-46173-0)
Supplement: Supplementary file 1 — Supplementary Methods [file 41598_2019_46173_MOESM1_ESM.pdf]

Does the endometrial cavity have a molecular microbial signature?

Andrew D. Winters[1,2,3], Roberto Romero[3-7*], Maria Teresa Gervasi[8], Nardhy Gomez-Lopez[1-3,9], Maria Rosa Tran[8], Valeria Garcia-Flores[3,9], Percy Pacora[3,9], Eunjung Jung[3,9], Sonia S. Hassan[2,3,9,10], Chaur-Dong Hsu[2,3,9,10], Kevin R. Theis[1,2,3,*]

[1] Department of Biochemistry, Microbiology, and Immunology, Wayne State University School of Medicine, Detroit, Michigan, USA

[2] Perinatal Research Initiative in Maternal, Perinatal and Child Health, Wayne State University School of Medicine, Detroit, Michigan, USA

[3] Perinatology Research Branch, Division of Obstetrics and Maternal-Fetal Medicine, Division of Intramural Research, *Eunice Kennedy Shriver* National Institute of Child Health and Human Development, National Institutes of Health, U.S. Department of Health and Human Services, Bethesda, Maryland, and Detroit, Michigan, USA

[4] Department of Obstetrics and Gynecology, University of Michigan, Ann Arbor, Michigan, USA

[5] Department of Epidemiology and Biostatistics, Michigan State University, East Lansing, Michigan, USA

[6] Center for Molecular Medicine and Genetics, Wayne State University, Detroit, Michigan, USA

[7] Detroit Medical Center, Detroit, Michigan, USA

[8] Department of Women's and Children's Health, University Hospital of Padua, Padua, Italy

[9] Department of Obstetrics and Gynecology, Wayne State University School of Medicine, Detroit, Michigan, USA

[10] Department of Physiology, Wayne State University School of Medicine, Detroit, Michigan, USA

***Address correspondence to:**

Roberto Romero, MD, DMedSci (email: prbchiefstaff@med.wayne.edu) and Kevin R. Theis, PhD (email: ktheis@med.wayne.edu)

SUPPLEMENTARY METHODS

Microbial profiles did not differ between paired mid-endometrial and whole-length endometrial samples

An analysis, using the touchdown PCR approach, was conducted to verify that the bacterial profiles of samples collected from the mid-endometrium were representative of paired samples collected from the whole-length of the endometrium (N = 9 subjects). 16S rRNA gene abundance, as determined through quantitative real-time PCR, did not differ between these paired sample types (paired t-test, p > 0.05). With respect to alpha diversity, neither the richness (Chao1) nor heterogeneity (Shannon, Inverse Simpson) of mid-endometrial and whole-length endometrial bacterial profiles differed (Wilcoxon matched pairs or paired t-tests, $p > 0.05$) **(Supplementary Methods Figure 1)**. With respect to beta diversity, the bacterial profiles of mid-endometrial and whole-length endometrial samples differed from those of background technical controls in both composition (PERMANOVA; Jaccard: mid-endometrial, F = 2.598, p = 0.0001; whole-length endometrial, F = 2.896, p = 0.0002) and structure (Bray-Curtis: mid-endometrial, F = 6.014, p = 0.0003; whole-length endometrial, F = 7.651, p = 0.0002) **(Supplementary Methods Figure 2)**. However, the bacterial profiles of these two endometrial sample types did not differ from each other in composition or structure (Jaccard: F = 0.954, p = 0.573; Bray-Curtis: F = 0.594, p = 0.784). Indeed, subject identity had far greater influence on the bacterial profiles of endometrial samples than whether the swab was taken of the mid- or whole-length endometrium (Jaccard: subject, R^2 = 0.49, p = 0.001, sample type, R^2 = 0.06, p = 0.16; Bray-Curtis: subject, R^2 =0.56, p = 0.001, sample type, R^2 = 0.49, p = 0.42) **(Supplementary Methods Figure 3)**.

Supplementary Methods Figure 1. Heat map illustrating similarity in percent relative abundances of prominent operational taxonomic units (≥ 1% average relative abundance) among mid-endometrial, whole-length endometrial, and DNA extraction kit samples. Amplification of 16S rRNA genes was performed using a touchdown PCR approach.

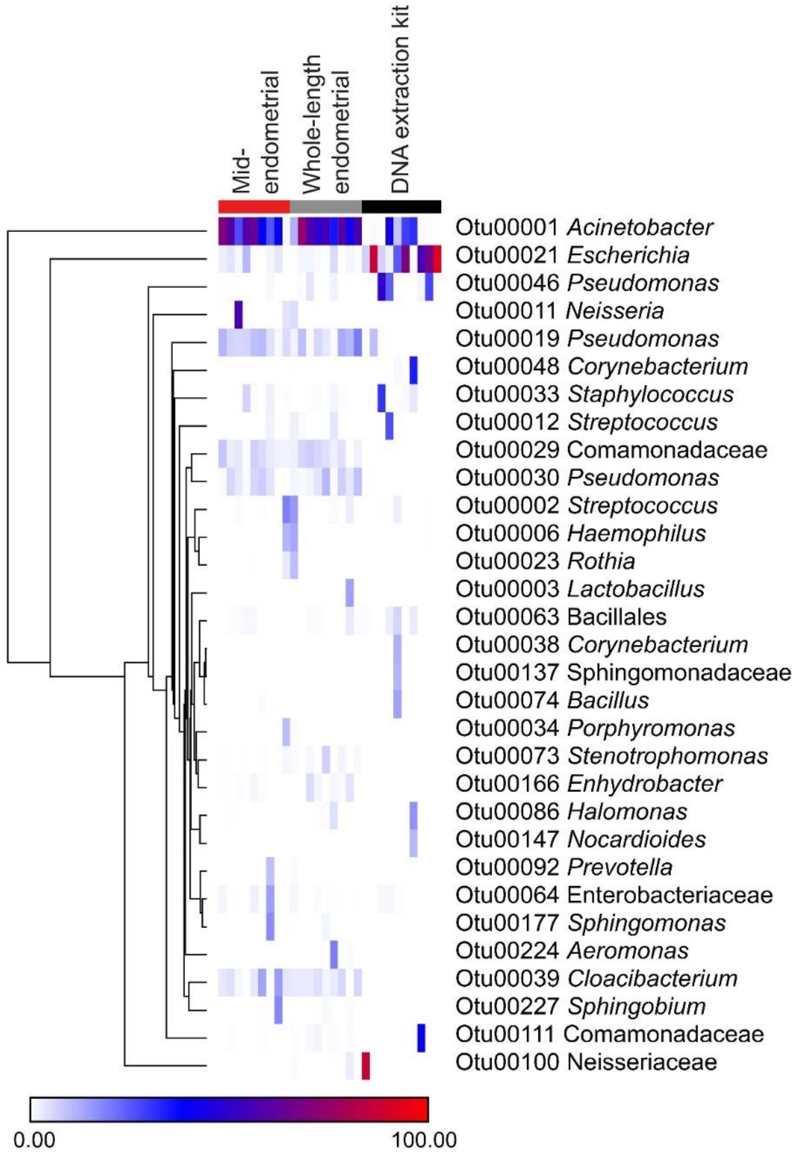

Supplementary Methods Figure 2. Principal Coordinates Analyses (PCoA) illustrating variation in 16S rRNA gene profile among mid-endometrial, whole-length endometrial, and DNA extraction kit samples. Profiles were generated for 16S rRNA gene community composition (a) and structure (b) using the Jaccard and Bray-Curtis indices, respectively.

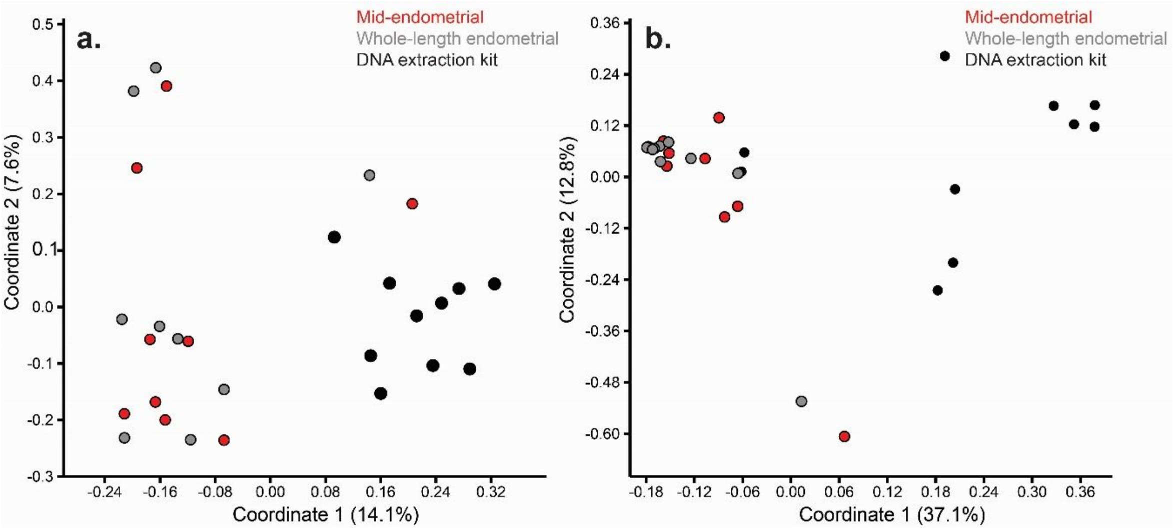

Supplementary Methods Figure 3. Principal Coordinates Analyses (PCoA) illustrating variation among subjects in 16S rRNA gene profiles of mid-endometrial (circle) and whole-length endometrial (triangle) samples. Symbol color indicates subject identity. Profiles were generated for 16S rRNA gene community composition (a) and structure (b) using the Jaccard and Bray-Curtis indices, respectively.

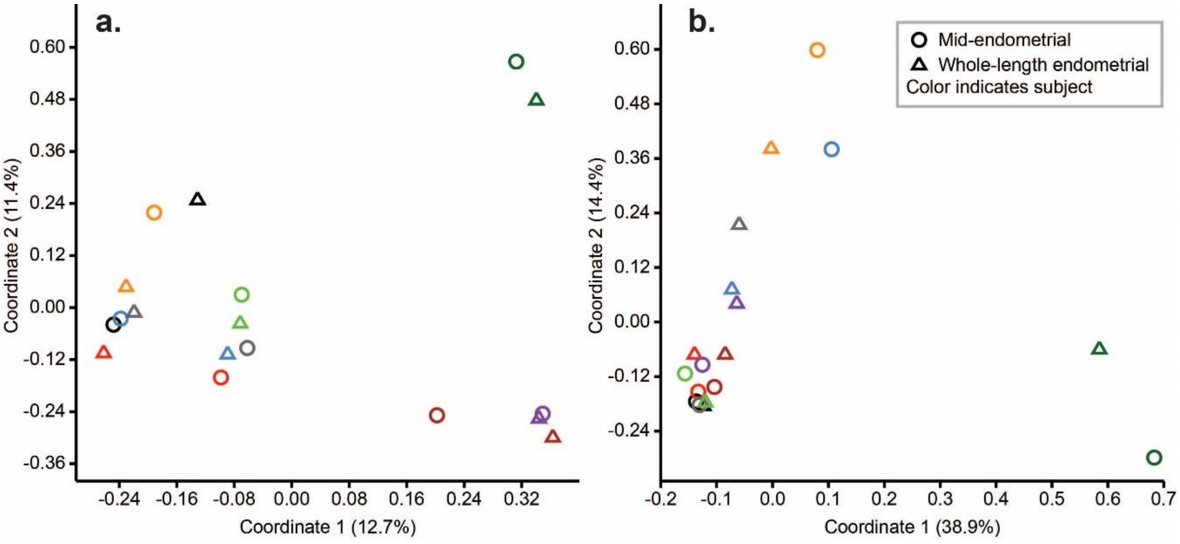

SUPPLEMENTARY TABLES

Supplementary Table S1. Description of prior 16S rRNA gene studies of the human endometrium

Study	Central research questions	Type of samples	Molecular microbiology methods	Relatively abundant / prevalent bacterial taxa identified in the endometrium	Were DNA contamination controls included?	Conclusions
Mitchell et al 2015[1]	Evaluate the presence of vaginal bacterial taxa in the upper genital tract of women undergoing hysterectomy for non-cancerous conditions.	Swabs of the vagina, upper endocervix, and the endometrium (N = 58).<br><br>Endometrial swabs were obtained post-hysterectomy.	Species-specific (12 vaginal bacterial species) and broad-range 16S rRNA gene qPCR.<br><br>Culture was also performed in a subset of 30 women.	*L. iners*, *Prevotella* spp., and *L. crispatus* were identified in one-third to one-half of the upper genital tracts through qPCR.<br><br>Diptheroids (corynebacteria), Gram-positive anaerobic cocci, *Propionibacterium*, and *Lactobacillus* were most commonly cultured from the upper genital tract.	Not reported.	95% of women had low levels of bacterial colonization in the endometrium and/or the upper endocervix (i.e. the upper genital tract) as determined by species-specific or broad-range qPCR.<br><br>87% of women were culture positive.
Fang et al 2016[2]	Characterize the intrauterine microbiota in healthy donors and women with endometrial polyps (with or without chronic endometritis).	Swabs of the vagina and endometrium (N = 10 healthy donors, and 20 women with endometrial polyps).<br><br>Endometrial swabs were obtained transcervically.	16S rRNA gene sequencing.	Overall, uterine bacterial profiles were dominated by *Lactobacillus*, followed by *Enterobacter* and *Pseudomonas*.<br><br>Among healthy women, the endometrial microbiota was dominated by *Enterobacter* and *Pseudomonas.*<br><br>Among women with endometrial polyps and chronic endometritis, the endometrial microbiota was dominated by *Lactobacillus*.	Not reported.	All women had an endometrial microbiota. The uterine microbiota of healthy donors and women with endometrial polyps differed.
Franasiak et al 2016[3]	Characterize the endometrial microbiota at the time	IVF catheter tip (N = 33).	16S rRNA gene sequencing.	*Lactobacillus* and *Flavobacterium* were most prevalent and relatively	"Positive controls utilizing *E. coli* along with negative	The endometrial microbiotas of successful and unsuccessful IVF patient groups did not differ.

	of embryo transfer by pregnancy outcome.			abundant.	controls were run to detect any contamination from reagents."<br><br>"The positive and negative controls for the study protocol were performed as expected."<br><br>Taxonomic assignment of any sequences from negative controls were not reported.	
Khan et al 2016[4]	Assess microbial colonization of the uterus and cystic fluid of women with endometriosis and asymptomatic control women with uterine myoma (with and without gonadotropin-releasing hormone agonist (GnRHa) treatment).	Endometrial swabs collected transcervically (N = 64; 32 with endometriosis; 32 with uterine myoma without endometriosis).<br><br>Cystic fluid was collected from women with (N = 8) and without (N = 8) ovarian endometrioma through laparoscopy.	16S rRNA gene sequencing.<br><br>Cystic fluids were cultured.	Predominant bacteria were Lactobacillacae, Streptococcaceae, Staphylococaceae, Enterobacteriaceae, and Moraxellaceae.	Not reported.	Streptococcaceae and Moraxellaceae were more relatively abundant among women with endometriosis.<br><br>Among women with endometriosis, Lactobacillaceae was decreased, and Streptococcaceae, Staphylococcaceae, and Enterobacteriaceae were increased, with GnRHa treatment.<br><br>Among women without endometriosis, Staphylococcaceae was increased with GnRHa treatment.<br><br>Streptococcaceae and Staphylococcaceae were increased, and Lactobacillaceae decreased, among women with ovarian endometrioma.<br>Culture of cystic fluid was negative.

Moreno et al 2016[5]	Investigate the existence of an endometrial microbiota in relation to that of the vagina, assess its hormonal regulation, and determine its effect on reproductive outcome in women undergoing IVF.	Endometrial fluid was obtained transcervically from 13 fertile women in perceptive and receptive phases of the menstrual cycle. Vaginal fluids were also obtained.<br><br>Secondarily, endometrial fluid was obtained from 22 fertile women in perceptive and receptive phases.<br><br>Lastly, endometrial fluids were obtained from 35 infertile women undergoing IVF.	16S rRNA gene sequencing.	The endometrial microbiota was dominated by *Lactobacillus*.<br><br>*Gardnerella*, and *Bifidobacterium* were also relatively abundant.	Not reported.	There is an endometrial microbiota.<br><br>Endometrial and vaginal microbiotas differed for some subjects.<br><br>The endometrial microbiota did not change in structure during the acquisition of endometrial receptivity.<br><br>Non-*Lactobacillus*-dominated microbiota was associated with significant decreases in implantation, ongoing pregnancy, and live birth rates.
Verstraelen et al 2016[6]	Evaluate the presence of a uterine microbiota in non-pregnant women with idiopathic reproductive conditions.	Endometrial brush samples were obtained transcervically (N = 19).	16S rRNA gene sequencing.	90% of women had endometrial bacterial profiles in which three *Bacteroides* species and one *Pelomonas* species accounted for over one third of the total.<br><br>There was an abundance of *Lactobacillus* in some subjects.	Not reported.	The data are consistent with the existence of a distinct endometrial microbiota.
Walther-Antonio et al 2016[7]	Compare intrauterine microbiota composition between women with and without endometrial cancer.	Swabs and scrapes from the vagina and cervix were taken pre-hysterectomy from women with benign	16S rRNA gene sequencing.<br><br>A microbial DNA enrichment kit	Endometrial samples were dominated by *Shigella* and *Barnesiella*.	Controls for both the DNA extraction and microbial enrichment processes were included and sequenced.	Significant subject-specific correlations in microbiota structure were observed across all organs.<br><br>The data suggest *Atopobium vaginae* and a *Porphyromonas*

		gynecologic conditions (N = 10), endometrial hyperplasia (N = 4), and endometrial cancer (N = 17).<br><br>Biopsies from the uterus, fallopian tube, and ovary were taken following hysterectomy.<br><br>Urine and stool samples were also collected.	was used to separate microbial DNA from human DNA prior to amplification for some samples (mostly tissues) that did not amplify.		Nine out of 14 controls yielded sequence data.<br><br>Relatively abundant taxa in controls included Enterobacteriaceae, *Methylobacterium*, *Moryella*, and *Staphylococcus*.<br><br>Contamination during sample collection was assessed using an open Petri dish containing Lysogeny broth.	spp. in the gynecologic tract are associated with endometrial cancer.
Chen et al 2017[8]	Investigate the presence of a microbiota in the upper reproductive tract and identify potential biomarkers of common reproductive tract diseases.	Swabs of the vagina and cervix of women with benign, non-infectious gynecological conditions (N = 110) were taken pre-hysterectomy.<br><br>Swabs of the endometrium, fallopian tubes, and peritoneal fluid were taken following hysterectomy.	16S rRNA gene sequencing.<br><br>Real-time quantitative PCR using primers targeting four vaginal *Lactobacillus* species.	Relative abundances of *Acinetobacter*, *Pseudomonas*, *Morganella*, *Sphingobium*, and *Vagococcus* increased from the lower reproductive tract to the upper reproductive tract, while relative abundances of *Lactobacillus* species decreased.<br><br>In the endometrium, while high relative abundances of *Lactobacillus* were detected, high relative abundances of *Pseudomonas*, *Acinetobacter*, *Vagococcus*, *Sphingobium*, and *Comamonadaceae* were also detected.	Sequence data for negative controls are publicly available.	An intra-individual continuum of microbiota along the female reproductive exists, and it is indicative of a non-sterile endometrium.
Miles et al	Investigate the presence of bacteria	With the exception of	16S rRNA gene	High relative abundances of *Lactobacillus* were detected	"Quality assurance and control of the	Bacteria were identified in 95% of samples.

2017[9]	throughout the reproductive tract of women undergoing a total hysterectomy and bilateral salpingo-oopherectomy.	vaginal swabs, swabs of the endometrium, cervix, myometrium, fallopian tube, and ovary were collected post-hysterectomy (N = 10).	sequencing.	in the endometrium in half of the women.<br><br>Increased relative abundances of *Acinetobacter* and *Corynebacterium* were observed for cervical and endometrial samples.	reactions were performed with both positive and negative control samples to ensure fidelity of the reagents and lack of contamination."<br><br>There was no report of the controls being sequenced.	The upper reproductive tract is not sterile in most women.<br><br>The structure of the microbiota in multiple sites is similar within a given woman.
Tao et al 2017[10]	Characterize the endometrial microbiota of women undergoing IVF.	IVF catheter tip (N = 70).	16S rRNA gene sequencing.	*Lactobacillus* was detected in all samples, with greater than 90% relative abundance in 33/70 samples, and greater than 50% relative abundance in 50/70 samples.<br><br>Other vaginal bacteria (*Bifidobacterium*, *Corynebacterium*, *Staphylococcus*, and *Streptococcus*) were also detected.	Varying concentrations of mock communities were used to validate that poly-microbial samples can be identified by the 16S rRNA gene sequencing assay performed.<br><br>One blank extraction control was sequenced. The most abundant taxa in the control sample were *Ralstonia*, *Pseudomonas*, *Cupriavidis*, *Agrobacterium*, *Mesorhizobium*, and *Hyphomicrobium*.	There is an endometrial microbiota.<br><br>Preamplification of raw lysates prior to 16S rRNA gene sequencing provides a sensitive approach for characterizing the endometrial microbiota.
Kyono et al 2018[11]	Assess variation in the endometrial microbiota among healthy volunteers, IVF patients, and non-IVF patients.	Endometrial fluid was collected transcervically with an intrauterine insemination catheter from IVF patients (N = 79), non-IVF patients (N = 23),	16S rRNA gene sequencing.	Endometrial samples from all were largely dominated by *Lactobacillus*. IVF patients also had endometrial microbiota containing high relative abundances of *Gardnerella*, *Streptococcus*, *Atopobium*, *Bifidobacterium*, *Sneathia*, *Prevotella*, and	One blank DNA extraction kit was sequenced.<br><br>"Blank-characteristic OTUs," including *Acinetobacter*, *Escherichia*, *Flavobacterium*, *Janthinobacterium*,	The percentages of *Lactobacillus* in the endometrium of IVF patients, non-IVF patients, and healthy volunteers were different.<br><br>62% of IVF patients have an endometrial microbiota that is not *Lactobacillus*-dominated.

		and healthy volunteers (N = 7). Swabs of vaginal discharge were also collected.		*Staphylococcus*.	*Methylobacterium*, *Pseudomonas*, *Rhodococcus*, *Sphingomonas*, and *Stenotrophomonas* were removed from the dataset prior to analysis.	
Liu et al 2018[12]	Assess the difference between microbiotas of endometrial tissue and fluid in IVF patients.	Paired endometrial fluid (lavage water) and tissue (biopsy) samples collected transcervically from 25 women with recurrent miscarriages.	16S rRNA gene sequencing.	Relatively abundant taxa in both tissue and fluid include: *Lactobacillus*, *Stenotrophomas*, *Gardnerella*, *Bifidobacterium*, *Atopobium*, *Prevotella*, *Megasphaera*, *Staphylococcus*, and *Escherichia*.	Negative controls included RNase- and DNase-free water used for rinsing the tissue and uterine cavity (N = 8), and swabs exposed to the air (N = 8).<br><br>Only two controls yielded sequences (6 and 12 reads). The taxonomic data were not reported.	There is an endometrial microbiota.<br><br>The composition of the microbiota in endometrial fluid is not completely reflective of that in endometrial tissue.<br><br>"Further efforts are needed to identify the preanalytical effects, including sampling sites, methods, and sequencing depth, on profiling endometrial microbiota."
Moreno et al 2018[13]	Is real-time polymerase chain reaction comparable to the use of histology, hysteroscopy, and/or microbial culture to diagnose chronic endometritis?	Endometrial biopsies obtained transcervically from women suspected of having chronic endometritis (N = 113).<br><br>95 biopsies yielded sufficient DNA for analysis.	Species-specific (9 potential endometritis agents) qPCR.<br><br>16S rRNA gene sequencing on 13 biopsies with confirmed chronic endometritis.<br><br>Culture was performed in a subset of 65 women.	Streptococci were most commonly identified through targeted qPCR.<br><br>*Enterococcus*, *Streptococcus*, and *Escherichia* were most often recovered in culture.<br><br>The 16S rRNA gene profiles of women with confirmed chronic endometritis were dominated by *Lactobacillus*, *Streptococcus*, and *Gardnerella*.	The qPCR assays included robust controls.<br><br>For 16S rRNA gene sequencing, "Positive controls of *E. coli* DNA and negative controls were included to detect any contamination from reagents." It was not reported whether these controls were sequenced.	56% of women tested for chronic endometritis were qPCR-positive for at least one endometritis agent.<br><br>52% of women were culture positive.<br><br>qPCR can be an inexpensive and rapid diagnostic tool for identifying chronic endometritis.
Pelzer et al 2018[14]	Characterize the endometrial and	Paired endometrial	16S rRNA gene	The endometrial and endocervical microbiotas of	Not reported.	There is an endometrial microbiota.

	endocervical microbiota in women with menorrhagia or dysmenorrhea.	curettings and endocervical swabs were collected transcervically from women with menorrhagia (N = 25), dysmenorrhea (N = 32), and virgo intacta controls (N = 3).	sequencing.	women with dysmenorrhea and menorrhagia were largely dominated by *Lactobacillu*s, with *Gardnerella*, *Veillonella*, *Prevotella*, and *Sneathia* also being abundant.<br><br>*Propionibacterium*, *Staphylococcus*, *Pseudomonas*, *Ralstonia*, *Corynebacterium*, and *Kocuria* were more relatively abundant in the endometrium than the endocervix.<br><br>*Jonquetella* and *Fusobacterium* were dominant in the endometrium of virgo intacta women.		The microbiotas of the endometrium and endocervix do not differ overall.<br><br>The endometrial microbiotas of women with menorrhagia and dysmenorrhea do not differ.
Wee et al 2018[15]	Compare the vaginal, cervical and endometrial microbiotas of women with a history of infertility and those with a history of fertility.	Vaginal swabs, endocervical swabs, and endometrial biopsies were collected transcervically from women with a history of infertility (N = 15) and women without infertility (N = 16).	16S rRNA gene sequencing.<br><br>RT-qPCR was conducted to detect *Ureaplasma* spp.<br><br>RT-qPCR of selected human gene transcripts in 2 endometrial tissues: (IL-1α, IL-6, IL-8, Tenascin-C, TNF α, and Syndecan 1).	*Lactobacillus* was the most common and relatively abundant taxon in vaginal, cervical, and endometrial samples.	Samples with low DNA yield were not sent for sequencing.<br><br>Negative controls for lysis, extraction, and PCR were sequenced and analyzed. They had a low sequence yield. Taxa were not reported.	Endometrial samples did not consistently yield sequence libraries.<br><br>When they did, endometrial and vaginal microbiotas did not consistently differ.<br><br>Expression of selected human genes in the endometrium did not correlate with either fertility status or microbiota composition.

Supplementary Table S2. Differences in alpha diversity values among paired body site samples for the standard PCR dataset based on three metrics (Chao 1 richness estimator, Shannon diversity index, and the inverse Simpson index). Differences were evaluated using linear mixed-effect models and ANOVA tests, controlling for subject (i.e., patient identity) as a random effect. OS = oral, RS = rectal, VS = vaginal, CS = cervical, and EMS = endometrial.

Chao ~ Type + (1|Subject)

	Sum Sq	Mean Sq	NumDF	DenDF	F value	Pr(>F)
Type	108582.9	27145.73	4	72.97271	44.95605	5.52E-19

	Estimate	Std. Error	z value	Pr(>|z|)
EMS-CS	6.120	11.893	0.515	1.000
OS-CS	32.721	7.790	4.201	< 0.0001
RS-CS	76.073	7.790	9.766	< 0.0001
VS-CS	-9.508	7.790	-1.221	1.000
OS-EMS	26.601	11.450	2.323	0.202
RS-EMS	69.953	11.450	6.110	< 0.0001
VS-EMS	-15.628	11.450	-1.365	1.000
RS-OS	43.352	6.950	6.237	< 0.0001
VS-OS	-42.229	6.950	-6.076	< 0.0001
VS-RS	-85.581	6.950	-12.313	< 0.0001

summary(glht(Standard_Chao_model, linfct = mcp(Type = "Tukey")), test = adjusted("bonferroni"))

lmer (Shannon ~ Type + (1|Subject)

	Sum Sq	Mean Sq	NumDF	DenDF	F value	Pr(>F)
Type	98.34393	24.58598	4	76.67927	63.90796	1.17E-23

	Estimate	Std. Error	z value	Pr(>|z|)
EMS-CS	-0.130	0.298	-0.438	1.000
OS-CS	1.303	0.196	6.650	< 0.0001
RS-CS	1.904	0.196	9.715	< 0.0001
VS-CS	-0.582	0.196	-2.969	0.030
OS-EMS	1.433	0.286	5.009	< 0.0001
RS-EMS	2.034	0.286	7.108	< 0.0001
VS-EMS	-0.451	0.286	-1.577	1.000
RS-OS	0.601	0.175	3.423	0.006
VS-OS	-1.885	0.175	-10.743	< 0.0001
VS-RS	-2.485	0.175	-14.167	< 0.0001

summary(glht(Standard_Shannon_model, linfct = mcp(Type = "Tukey")), test = adjusted("bonferroni"))

lmer (InvSimpson ~ Type + (1|Subject)

	Sum Sq	Mean Sq	NumDF	DenDF	F value	Pr(>F)
Type	6921.09	1730.273	4	69.97894	35.71063	3.07E-16

	Estimate	Std. Error	z value	Pr(>|z|)
EMS-CS	-0.569	3.306	-0.172	1.000
OS-CS	11.481	2.188	5.246	< 0.0001
RS-CS	19.345	2.188	8.840	< 0.0001
VS-CS	-0.681	2.188	-0.311	1.000
OS-EMS	12.049	3.165	3.807	0.001
RS-EMS	19.914	3.165	6.293	< 0.0001
VS-EMS	-0.112	3.165	-0.035	1.000
RS-OS	7.864	1.969	3.994	0.001
VS-OS	-12.161	1.969	-6.177	< 0.0001
VS-RS	-20.026	1.969	-10.171	< 0.0001

summary(glht(Standard_InvSimpson_model, linfct = mcp(Type = "Tukey")), test = adjusted("bonferroni"))

Supplementary Table S3. Differences in alpha diversity values between cervical, endometrial, and background technical control samples for the touchdown PCR dataset based on three metrics (Chao 1 richness estimator, Shannon diversity index, and the inverse Simpson index). Differences were evaluated using Mann-Whitney/Wilcoxon rank-sum tests. CS = cervical, EMS = endometrial, and BLK = technical background control.

		Sum of ranks	*z* value	Effect size	*p* value
Chao 1	CS_EMS	17.0	-1.379	0.308	0.168
	CS_BLK	79.5	-0.480	0.107	0.631
	EMS_BLK	60.0	0.000	0.000	1.000
Shannon	CS_EMS	16.0	-1.467	0.328	0.142
	CS_BLK	151.0	-2.901	0.649	0.004
	EMS_BLK	104.0	-2.868	0.641	0.004
Inverse Simpson	CS_EMS	21.0	-1.022	0.229	0.307
	CS_BLK	131.0	-1.942	0.434	0.052
	EMS_BLK	91.0	-2.011	0.450	0.044

Supplementary Table S4. Genera indicated by Linear discriminant analysis Effect Size (LEfSe) as being more relatively abundant in the endometrium than in background technical controls

Genus	Ecological and clinical description of the genus and its reported occurrence in prior sequence-based studies of the human endometrium	Has this genus been documented as a DNA contaminant in prior sequence-based studies?
Acinetobacter	A diverse genus containing both common soil and clinically relevant bacteria that can cause a range of opportunistic, often catheter-related, infections in humans[16].<br><br>*Acinetobacter* was identified at low relative abundances (i.e., < 1%) in seven endometrial microbiota studies[2,3,6,7,10,12,14], and was present at varying abundances (i.e., 5 - 30% in some samples) in others[5,8,9,13].	Yes[10,12,17,18]
Pseudomonas	A diverse group of bacteria that inhabit a wide variety of environments and can colonize many different mucosal surfaces, invade tissues and blood, and cause nosocomial infections[19,20].<br><br>*Pseudomonas* was identified at low relative abundances in six endometrial microbiota studies[5-7,10,12,14], and at abundances of ≥ 5% in three others[2,3,8].	Yes[7,10,12,17,18]
Cloacibacterium	A genus with species previously isolated from wastewater[21], freshwater lake sediment[22], activated sludge[23], and the intestinal tract of a bivalve[24]. Using 16S rDNA sequencing, *C. normanense* was detected in a tissue sample of a patient with spondylodiscitis[25].<br><br>*Cloacibacterium* was identified in one endometrial microbiota study at a low relative abundance[5].	Yes[18]
Haemophilus	A diverse genus containing strains that cause pathogenic infection in both animals and humans. *Haemophilus* species can be commensals of the mucous membranes[26].<br><br>*Haemophilus* was identified in four endometrial microbiota studies at low relative abundances[5,6,9,13].	Yes[18]
Flavobacterium	The genus has more than 100 species of commensal bacteria and opportunistic pathogens of freshwater fish that are common in sediments and aquatic environments[27,28]. *F. lindanitolerans* was isolated from the ascites of a patient in China with Enterovirus 71 infection who died of fatal pulmonary edema and hemorrhage[29].	Yes[17]

	Flavobacterium was identified in two endometrial microbiota studies at low relative abundances[12,13].	
Veillonella	Common commensals found in the alimentary canal and vagina of mammals that are often associated with bite wounds and infections of the mouth, sinuses, lungs, heart, bone, and central nervous system[30].<br><br>*Veillonella* was identified in six endometrial microbiota studies at low relative abundances[2,5,7,9-11].	Yes[7,18]
Stenotrophomonas	Bacteria commonly isolated from, sewage, sludge, and soil that can be agents of nosocomial infections[31], especially among immunocompromised patients[32,33].<br><br>*Stenotrophomonas* was identified at low relative abundances in eight endometrial microbiota studies [2,5-10,12].	Yes[17,18]
Enhydrobacter	This genus has a single environmental species[34].<br><br>*Enhydrobacter* was identified at low relative abundances in three endometrial microbiota studies[2,5,8].	Yes[7,17,18]
Fusobacterium	This genus contains several species that inhabit the mucous membranes of humans and animals[35]. The presence of *Fusobacterium* is associated with periodontitis[36], thrombophlebitis[37], and colorectal carcinoma[38].<br><br>*Fusobacterium* was identified in seven endometrial microbiota studies at low relative abundances[2,5-8,10,14].	Yes[7,18,39]
Actinomyces	Ubiquitous bacteria found in soil and in the microbiota of animals; they can be opportunistic pathogens[40].<br><br>*Actinomyces* was identified in five endometrial microbiota studies at low relative abundances [2,5-7,10].	Yes[7,18]

SUPPLEMENTARY FIGURES

Supplementary Figure S1. Alpha diversity values based on three metrics (Chao 1 richness estimator, Shannon diversity index, and the inverse Simpson index) for 16S rRNA gene profiles of the five body sites for the standard PCR dataset and of the cervical, endometrial, and technical control samples for the touchdown PCR dataset.

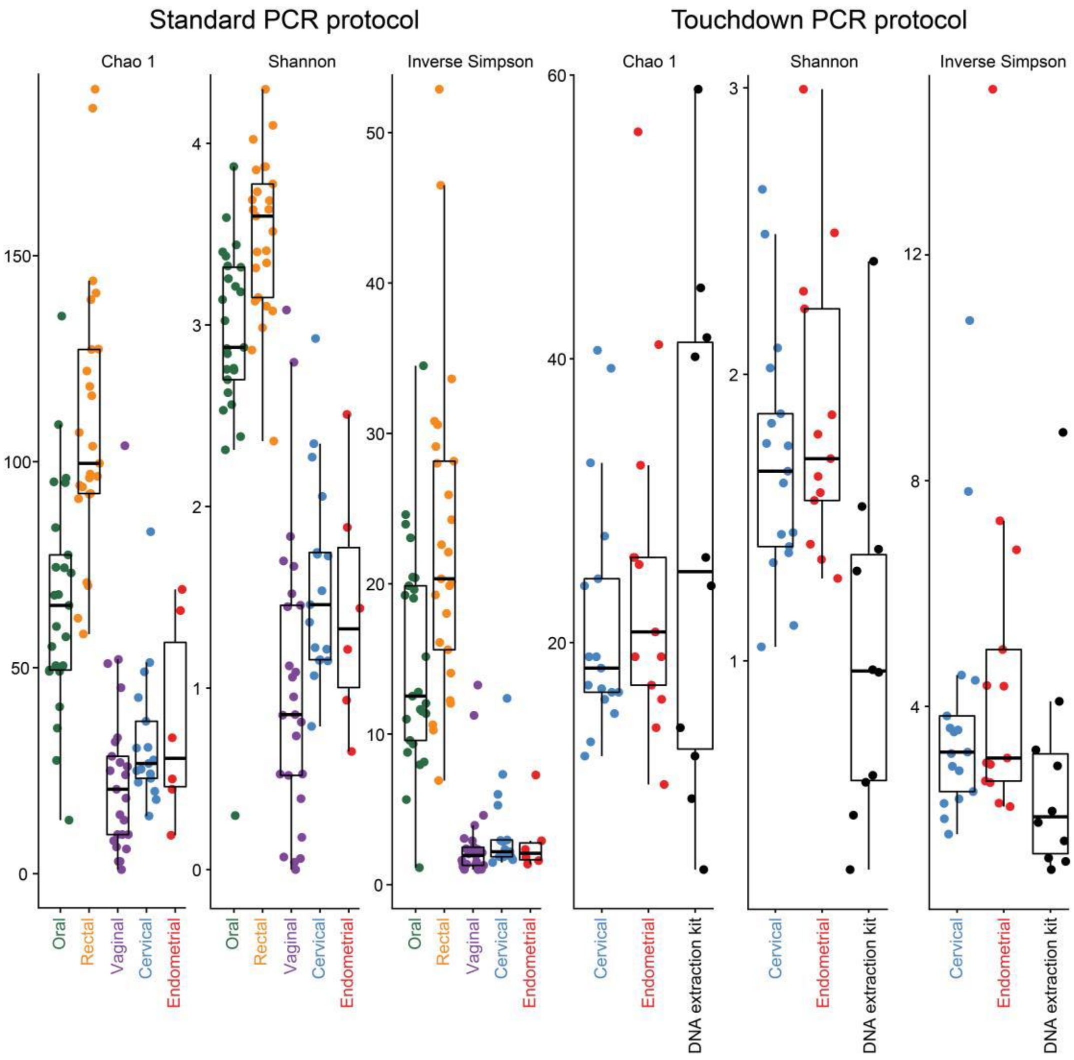

Supplementary Figure S2. Heat map illustrating percent relative abundances of amplicon sequence variants among cervical, endometrial, and background technical control samples. Amplification of 16S rRNA genes was performed using both standard PCR and touchdown PCR approaches. Each amplicon sequence variant differed at most by one base pair (bp) from the consensus sequence of its respective operational taxonomic unit (OTU) (a-e).

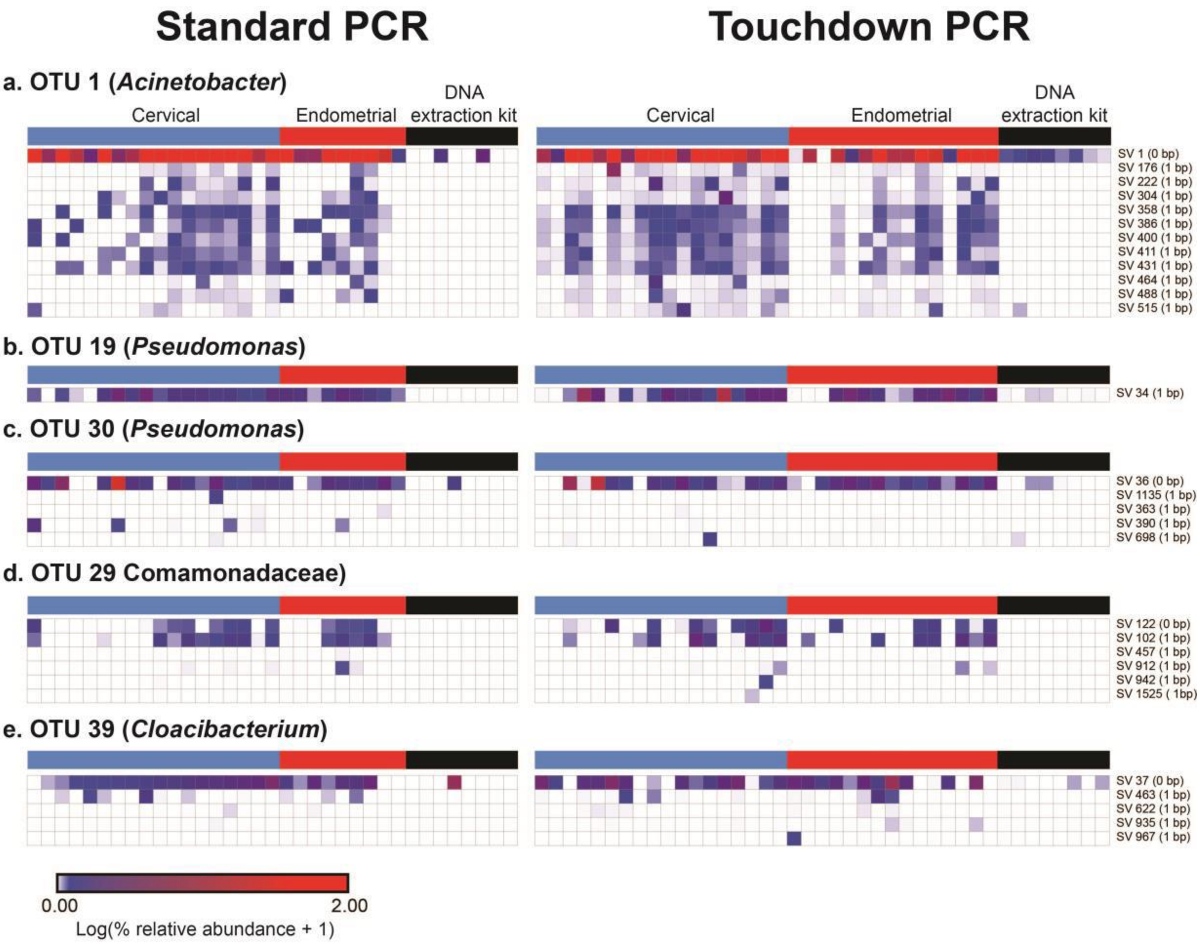

REFERENCES

1 Mitchell, C. M. *et al.* Colonization of the upper genital tract by vaginal bacterial species in nonpregnant women. *Am J Obstet Gynecol* **212**, 611.e611-619 (2015).

2 Fang, R. L. *et al.* Barcoded sequencing reveals diverse intrauterine microbiomes in patients suffering with endometrial polyps. *Am J Transl Res* **8**, 1581-1592 (2016).

3 Franasiak, J. M. *et al.* Endometrial microbiome at the time of embryo transfer: next-generation sequencing of the 16S ribosomal subunit. *J Assist Reprod Genet* **33**, 129-136 (2016).

4 Khan, K. N. *et al.* Molecular detection of intrauterine microbial colonization in women with endometriosis. *Eur J Obstet Gynecol Reprod Biol* **199**, 69-75 (2016).

5 Moreno, I. *et al.* Evidence that the endometrial microbiota has an effect on implantation success or failure. *Am J Obstet Gynecol* **215**, 684-703 (2016).

6 Verstraelen, H. *et al.* Characterisation of the human uterine microbiome in non-pregnant women through deep sequencing of the V1-2 region of the 16S rRNA gene. *PeerJ* **4**, e1602 (2016).

7 Walther-Antonio, M. R. *et al.* Potential contribution of the uterine microbiome in the development of endometrial cancer. *Genome Med* **8**, 122 (2016).

8 Chen, C. *et al.* The microbiota continuum along the female reproductive tract and its relation to uterine-related diseases. *Nat Commun* **8**, 875 (2017).

9 Miles, S. M., Hardy, B. L. & Merrell, D. S. Investigation of the microbiota of the reproductive tract in women undergoing a total hysterectomy and bilateral salpingo-oopherectomy. *Fertil Steril* **107**, 813-820.e811 (2017).

10 Tao, X. *et al.* Characterizing the endometrial microbiome by analyzing the ultra-low bacteria from embryo transfer catheter tips in IVF cycles: Next generation sequencing (NGS) analysis of the 16S ribosomal gene. *Human Microbiome Journal* **3**, 15-21 (2017).

11 Kyono, K., Hashimoto, T., Nagai, Y. & Sakuraba, Y. Analysis of endometrial microbiota by 16S ribosomal RNA gene sequencing among infertile patients: a single-center pilot study. *Reprod Med Biol* **17**, 297-306 (2018).

12 Liu, Y. *et al.* Systematic comparison of bacterial colonization of endometrial tissue and fluid samples in recurrent miscarriage patients: Implications for future endometrial microbiome studies. *Clin Chem* **64**, 1743-1752 (2018).

13 Moreno, I. *et al.* The diagnosis of chronic endometritis in infertile asymptomatic women: a comparative study of histology, microbial cultures, hysteroscopy, and molecular microbiology. *Am J Obstet Gynecol* **218**, 602.e601 (2018).

14 Pelzer, E. S., Willner, D., Buttini, M. & Huygens, F. A role for the endometrial microbiome in dysfunctional menstrual bleeding. *Antonie Van Leeuwenhoek* **111**, 933-943(2018).

15 Wee, B. A. *et al.* A retrospective pilot study to determine whether the reproductive tract microbiota differs between women with a history of infertility and fertile women. *Aust N Z J Obstet Gynaecol* **58**, 341-348 (2018).

16 Towner, K. The genus *Acinetobacter* in *The Prokaryotes: Volume 6: Proteobacteria: Gamma Subclass* (ed Martin Dworkin) 746-758 (Springer, 2006).

17 Salter, S. J. *et al.* Reagent and laboratory contamination can critically impact sequence-based microbiome analyses. *BMC Biol* **12**, 87 (2014).

18 Glassing, A., Dowd, S. E., Galandiuk, S., Davis, B. & Chiodini, R. J. Inherent bacterial DNA contamination of extraction and sequencing reagents may affect interpretation of microbiota in low bacterial biomass samples. *Gut Pathog* **8**, 24 (2016).

19 Moore, E. R. *et al.* Nonmedical: pseudomonas in *The Prokaryotes: Volume 6: Proteobacteria: Gamma Subclass* (ed Martin Dworkin) 646-703 (Springer, 2006).

20 Yahr, T. L. & Parsek, M. R. *Pseudomonas aeruginosa* in *The Prokaryotes: Volume 6: Proteobacteria: Gamma Subclass* (ed Martin Dworkin) 704-713 (Springer, 2006).
21 Allen, T. D., Lawson, P. A., Collins, M. D., Falsen, E. & Tanner, R. S. *Cloacibacterium normanense* gen. nov., sp. nov., a novel bacterium in the family Flavobacteriaceae isolated from municipal wastewater. *Int J Syst Evol Microbiol* **56**, 1311-1316 (2006).
22 Cao, S. J., Deng, C. P., Li, B. Z., Dong, X. Q. & Yuan, H. L. *Cloacibacterium rupense* sp. nov., isolated from freshwater lake sediment. *Int J Syst Evol Microbiol* **60**, 2023-2026 (2010).
23 Chun, B. H., Lee, Y., Jin, H. M. & Jeon, C. O. *Cloacibacterium caeni* sp. nov., isolated from activated sludge. *Int J Syst Evol Microbiol* **67**, 1688-1692 (2017).
24 Hyun, D. W. *et al. Cloacibacterium haliotis* sp. nov., isolated from the gut of an abalone, *Haliotis discus hannai*. *Int J Syst Evol Microbiol* **64**, 72-77 (2014).
25 Stavnsbjerg, C., Frimodt-Moller, N., Moser, C. & Bjarnsholt, T. Comparison of two commercial broad-range PCR and sequencing assays for identification of bacteria in culture-negative clinical samples. *BMC Infect Dis* **17**, 233 (2017).
26 Hoiseth, S. K. The genus *Haemophilus* in *The Prokaryotes*: *Volume 4*: *A Handbook on the biology of Bacteria*: *Ecophysiology, Isolation, Identification, Applications* (ed Albert Balows) 3304-3330 (Springer, 1992).
27 Bernardet, J. & Bowman, J. The Genus *Flavobacterium* in *The Prokaryotes: Volume 7*: *Deeply Rooting Bacteria* (ed Martin Dworkin) 481-531 (Springer, 2006).
28 Loch, T. P. & Faisal, M. Emerging flavobacterial infections in fish: A review. *J Adv Res* **6**, 283-300 (2015).
29 Tian, G. Z. *et al.* A *Flavobacterium lindanitolerans* strain isolated from the ascites sample of a Chinese patient with EV71 virus infection. *Biomed Environ Sci* **24**, 694-696 (2011).
30 Kolenbrander, P. The genus *Veillonella* in *The Prokaryotes*: *Volume 4*: *A Handbook on the biology of Bacteria*: *Bacteria*: *Firmicutes, Cyanobacteria* (ed Martin Dworkin) 1022-1040 (Springer, 2006).
31 Palleroni, N. J. & Bradbury, J. F. *Stenotrophomonas*, a new bacterial genus for *Xanthomonas maltophilia* (Hugh 1980) Swings et al. 1983. *Int J Syst Evol Microbiol* **43**, 606-609 (1993).
32 Calza, L., Manfredi, R. & Chiodo, F. *Stenotrophomonas* (*Xanthomonas*) *maltophilia* as an emerging opportunistic pathogen in association with HIV infection: a 10-year surveillance study. *Infection* **31**, 155-161 (2003).
33 Safdar, A. & Rolston, K. V. *Stenotrophomonas maltophilia*: changing spectrum of a serious bacterial pathogen in patients with cancer. *Clin Infect Dis* **45**, 1602-1609 (2007).
34 Staley, J. T., Irgens, R. L. & Brenner, D. J. *Enhydrobacter aerosaccus* gen. nov., sp. nov., a gas-vacuolated, facultatively anaerobic, heterotrophic rod. *Int J Syst Evol Microbiol* **37**, 289-291 (1987).
35 Hofstad, T. The genus *Fusobacterium* in *The Prokaryotes*: *Volume 4*: *A Handbook on the biology of Bacteria*: *Ecophysiology, Isolation, Identification, Applications* (ed Albert Balows) 4114-4126 (Springer, 1992).
36 Signat, B., Roques, C., Poulet, P. & Duffaut, D. *Fusobacterium nucleatum* in periodontal health and disease. *Curr Issues Mol Biol* **13**, 25-36 (2011).
37 Weeks, D. F., Katz, D. S., Saxon, P. & Kubal, W. S. Lemierre syndrome: report of five new cases and literature review. *Emerg Radiol* **17**, 323-328 (2010).
38 Castellarin, M. *et al. Fusobacterium nucleatum* infection is prevalent in human colorectal carcinoma. *Genome Res* **22**, 299-306 (2012).
39 Kim, D. *et al.* Optimizing methods and dodging pitfalls in microbiome research. *Microbiome* **5**, 52 (2017).

40 Schaal, K. P., Yassin, A. F. & Stackebrandt, E. The family Actinomycetaceae: The genera *Actinomyces*, *Actinobaculum*, *Arcanobacterium*, *Varibaculum*, and *Mobiluncus* in *The Prokaryotes: Volume 3: Archaea. Bacteria: Firmicutes, Actinomycetes* (ed Martin Dworkin) 430-537 (Springer, 2006).
